# Polymeric and Chelate Gel Precursors for Transition Metal Oxide and Silicon-Based Anodes in Lithium–Ion Batteries

**DOI:** 10.3390/gels12060500

**Published:** 2026-06-04

**Authors:** Mobinul Islam, Md. Shahriar Ahmed, Yoomin Kim, Jemin Yeon, Jihun Kim, Ye-Chan Oh, Md. Mahmudul Hasan, Hyerim Hong, Yuchae Hwang, Kyung-Wan Nam

**Affiliations:** 1 Department of Energy & Materials Engineering, Dongguk University-Seoul, Seoul 04620, Republic of Korea; mobinsust@dongguk.edu (M.I.); shahriar.emcl@dgu.ac.kr (M.S.A.); 2024126678@dgu.ac.kr (Y.K.); redhrim@dgu.ac.kr (H.H.); dbco48@dgu.ac.kr (Y.H.); 2 Department of Advanced Battery Convergence Engineering, Dongguk University-Seoul, Seoul 04620, Republic of Korea; wpals7112@dgu.ac.kr (J.Y.); 2024126739@dgu.ac.kr (J.K.); dhdpcks0432@dgu.ac.kr (Y.-C.O.); 3 Research Organization for Nano and Life Innovation, Waseda University, Tokyo 162-0041, Japan; hasan@aoni.waseda.jp

**Keywords:** gel precursor, sol–gel, chelating agent, Si-based anode, polymeric gel, lithium–ion battery, anode material, transition metal oxide

## Abstract

The growing demand for efficient and sustainable energy storage systems has intensified research on advanced materials for lithium–ion batteries (LIBs). Gel-based synthesis routes—particularly polymeric and chelating gel techniques—have emerged as powerful methods for designing lithium–ion battery (LIB) anode materials with tailored microstructures, composition uniformity, and enhanced electrochemical performance. These methods facilitate the transformation of solution-phase precursors into homogeneous and finely structured materials, enabling precise tuning of physicochemical properties. This review provides a comprehensive overview of the fundamental principles of polymeric and chelate gel synthesis routes, highlighting their ability in controlling particle size, morphology, and phase purity. Their applicability to a wide range of anode materials, including transition metal oxides and silicon-based composites, is discussed. The manuscript highlights LIBs anode material developments via gel precursor chemistry, structure–property relationships, and future directions toward scalable and sustainable electrode manufacturing.

## 1. Introduction

Lithium–ion batteries (LIBs) anodes are crucial in shaping the energy density, rate capability, cost efficiency, and long-term cyclability of battery systems. LIBs are extensively used in smartphones, laptops, electric vehicles (EVs), and grid-scale storage due to their high energy efficiency, long cycle life, and lightweight design [1,2]. LIBs comprise several key components, with the anode material playing a significant role in achieving optimal battery performance. Commercial anodes are mainly made of graphite. They are widely used in lithium–ion batteries due to their stability, low cost, and efficient lithium–ion intercalation. However, graphite anodes have limited energy density and power capability, prompting research into alternative materials. Following their initial commercialization by Sony Corporation in the early 1990s [3], LIB performance has advanced significantly, with notable improvements in specific capacity, safety, and mechanical stability. A key driver behind this progress is the development of advanced cathode and anode materials with higher lithium storage capacity, such as lithium iron phosphate (LiFePO_4_), lithium nickel cobalt aluminum oxide (NCA), and lithium nickel cobalt manganese oxide (NCM), silicon/silicon-based composite anode, titanium-based compounds (known as zero-strain anode), and carbon-based hybrids, which offer superior volumetric and gravimetric capacities, thermal stability, and electrochemical performance compared to conventional LiCoO_2_ cathodes and graphite anodes [4,5,6,7].

While commercial graphite anodes offer excellent cyclability and safety, their limited theoretical capacity (372 mAh g^−1^) constrains further advancements in next-generation LIBs. Consequently, extensive research has focused on alternative anode materials, particularly transition metal oxides (TMOs) and silicon-based systems. TMOs (e.g., NiO, Fe_3_O_4_, TiO_2_, Co_3_O_4_, MnO_2_) operate through conversion or intercalation mechanisms, offering capacities in the range of 600–1000 mAh g^−1^, while silicon delivers an exceptionally high theoretical capacity (~4200 mAh g^−1^ for Li_15_Si_4_) [5,8,9]. However, both material classes face critical challenges, including large volume expansion, poor electrical conductivity, structural instability, and unstable solid–electrolyte interphase (SEI) formation, which hinder their practical implementation. To address these limitations, significant efforts have been devoted to engineering nanostructured and composite anode materials with controlled morphology, composition, and interface chemistry [10,11,12]. Those anode-active materials are typically synthesized using a wide range of wet-chemical and solid-state methods. Common synthesis techniques include sol–gel methods (gel-precursor based), hydrothermal/solvothermal synthesis, co-precipitation, combustion, spray pyrolysis, emulsion drying, and solid-state reactions [10,13,14,15,16,17,18,19,20,21,22]. Among various synthesis approaches, gel-based methods, particularly polymeric gels (e.g., the Pechini method) and chelate gels (e.g., the citric acid–nitrate route), have emerged as powerful strategies for designing advanced electrode materials with superior structural homogeneity and tunability. These methods rely on the formation of a three-dimensional polymeric or chelated network that uniformly distributes metal ions at the molecular level, followed by controlled thermal decomposition to yield highly dispersed, nanoscale oxide frameworks [20,21,22,23,24]. Such bottom-up synthesis enables precise control over stoichiometry, particle size, porosity, and phase purity, which are critical parameters governing lithium storage performance.

Polymeric gel routes typically involve the complexation of metal ions with organic ligands followed by polyesterification with polyols, such as ethylene glycol, to form a stable polymeric resin. This method ensures homogeneous mixing of multiple metal species and suppresses phase segregation during calcination, making it particularly suitable for synthesizing multicomponent TMOs and doped oxide systems [25,26]. In contrast, chelate gel methods utilize metal–ligand coordination chemistry, often combined with oxidizing agents (e.g., nitrates), to produce gel precursors that can undergo self-combustion or low-temperature calcination, yielding highly porous and nanostructured materials [27]. Both approaches offer distinct advantages, including low processing temperatures, scalability, compositional flexibility, and the ability to integrate carbonaceous or polymer-derived conductive networks in situ. In the context of silicon-based anodes, gel-derived strategies provide additional benefits by enabling the fabrication of silicon/oxide composites, carbon-coated silicon, and porous silicon frameworks that can effectively accommodate volume changes during lithiation/delithiation. The incorporation of polymeric or chelating agents not only facilitates the uniform dispersion of silicon particles but also promotes the formation of conductive carbon matrices upon pyrolysis, thereby enhancing electrical conductivity and structural integrity. Furthermore, gel-based synthesis enables the rational design of hybrid architectures, such as Si–TMO composites and yolk–shell structures, that can mitigate mechanical degradation and improve cycling stability [28,29,30,31,32].

This review aims to provide a comprehensive and critical overview of polymeric and chelate gel precursor strategies for the synthesis of transition-metal oxide and silicon-based anode materials for LIBs. We systematically discuss the fundamental chemistry of gel formation, processing parameters, and structure-property relationships, followed by an in-depth comparison of polymeric and chelate gel approaches. Particular emphasis is placed on how these methods influence particle morphology, porosity, phase evolution, and interfacial characteristics, and how these factors translate into electrochemical performance metrics such as capacity, rate capability, and cycle life. Unlike previous reviews, this work systematically compares polymeric and chelate gel routes for both TMO and Si-based anodes, with particular emphasis on morphology–performance relationships [10,11,12,13,14,15,16,17,18,19,20,21]. Finally, current challenges, scalability considerations, and future perspectives are highlighted to guide the rational design of next-generation gel-derived anode materials for high-performance lithium–ion batteries.

## 2. Overview of Gel-Based Precursor Chemistry

### 2.1. Sol–Gel Method

**Scheme 1:**
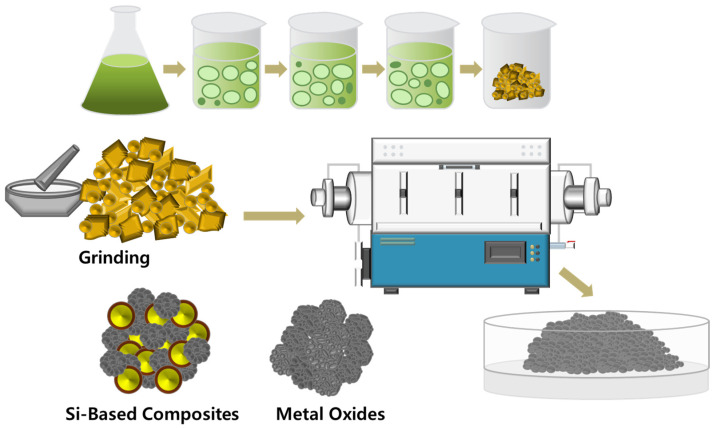
Schematic representation of the different steps in a traditional sol–gel synthesis process from starting materials to gel formation and final products via calcination.

The sol–gel process is a powerful wet-chemical technique widely used to synthesize metal oxide materials with tunable composition, morphology, and microstructure. The process begins with a stable “sol,” a colloidal solution of metal-based precursors, typically metal alkoxides (such as titanium isopropoxide) or inorganic metal salts (such as iron nitrates or chlorides). These precursors undergo a sequence of hydrolysis and condensation reactions in the presence of water, whether added directly or derived from environmental moisture. During hydrolysis, the metal-alkoxide bonds (M-OR) are replaced by hydroxyl groups (M-OH), which subsequently condense to form metal–oxygen–metal (M–O–M) linkages, resulting in a continuous, interconnected network that defines the gel phase [33,34,35]. This “wet gel” contains a large amount of solvent, which is then removed via drying methods (ambient, vacuum, or supercritical) to yield a porous “xerogel” or “aerogel”. Finally, a heat treatment (calcination) is applied to induce crystallization and remove any residual organics, producing the final Si-based composite or metal oxide materials, as shown in Scheme 1. Unlike conventional high-temperature solid-state methods, which rely on the physical mixing and diffusion of solid precursors at elevated temperatures (>1000 °C), the sol–gel approach allows for molecular-level mixing and reactions, ensuring exceptional chemical homogeneity at much lower synthesis temperatures (typically 300–700 °C) [34,35,36,37].

One of the key advantages of the sol–gel method is its ability to tailor the structural and morphological features of the resulting material with high precision. Since the process is solution-based, it facilitates the synthesis of a wide range of nanostructured materials, including nanoparticles, nanorods, mesoporous networks, and thin films [37,38]. For instance, by using titanium isopropoxide in alcohol–water mixtures, researchers can produce high-purity TiO_2_ nanoparticles with controlled size and crystalline phase (anatase or rutile), which are essential for photocatalytic and battery applications [39,40,41,42]. Similarly, Fe_3_O_4_ nanostructures can be obtained from iron nitrate or iron chloride precursors under appropriate pH and redox conditions, making them valuable for use in lithium–ion battery anodes, magnetic storage, and biomedical imaging [43,44]. The process also enables the integration of dopants or secondary phases uniformly within the matrix, allowing the synthesis of complex multicomponent oxides such as LiFePO_4_ or doped perovskites with improved electrochemical or catalytic behavior [45,46]. Additionally, the sol–gel method offers fine control over porosity and surface area, crucial for applications in catalysis, sensors, and energy storage, where surface accessibility directly impacts performance. Porous gels and aerogels, for example, can provide very high surface areas (several hundred m^2^/g), enhancing ion diffusion and reaction kinetics [33,36,38]. However, careful control of drying parameters is necessary to prevent shrinkage or cracking of the gel structure and scaling up sol–gel processing can be complex due to its sensitivity to ambient conditions and precursor stability [35]. Nonetheless, the sol–gel method remains a cornerstone in advanced materials chemistry, offering unmatched flexibility for designing functional oxides at the nanoscale with controlled phase, morphology, and purity.

The “traditional” sol–gel chemistry is, without a doubt, one of the most extensively studied and used fields of materials chemistry today. Despite this, alkoxide-based sol–gel routes suffer from inherent limitations. A key challenge is that many elements do not readily form stable alkoxides, thereby restricting the applicability of hydrolysis–condensation pathways [47]. In addition, these precursors are often highly reactive and moisture-sensitive, which complicates handling and limits compatibility with aqueous systems, particularly those involving water-soluble templates or structure-directing agents. Although careful control of reaction conditions can enable the use of such sensitive alkoxides, their practical versatility remains constrained [37,48]. To overcome these issues, alternative strategies based on aqueous metal salts have been developed. While these methods involve different underlying chemistries, they share the same objective of achieving controlled conversion of solution-phase precursors into metal oxide or ceramic materials. Broadly, these alternative sol–gel approaches can be classified into two main categories: chelate gel and organic polymeric gel, based on their distinct formation mechanisms [24,25,27,49,50].

### 2.2. Polymeric (Pechini) Method

The Pechini Method is a polymeric route to prepare uniform metal oxide anode materials. The Pechini method, also known as the polymerizable complex method, is a modified sol–gel process that enables the synthesis of highly uniform, stoichiometrically precise, and fine-grained metal oxides [50,51]. Initially developed by M.P. Pechini in the 1960s for ferroelectric ceramics, this approach has since gained significant traction in the field of energy storage materials, particularly for preparing anode materials for lithium–ion batteries (LIBs) [52]. Its inherent versatility and control over cation distribution make it exceptionally valuable for the synthesis of complex and doped transition metal oxides.

**Scheme 2:**
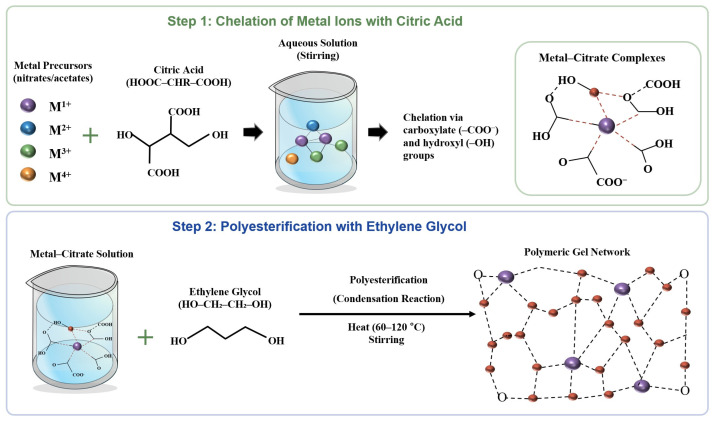
Schematic representation of chelation and polyesterification steps of the Pechini method.

The core mechanism of the Pechini method involves two key steps: chelation of metal ions using a hydroxycarboxylic acid such as citric acid, followed by polyesterification with a polyol like ethylene glycol. In the first stage, the metal precursors, typically nitrates or acetates, are mixed with citric acid in aqueous solution. Citric acid acts as a chelating agent, binding metal ions through its carboxylate and hydroxyl groups, effectively preventing premature precipitation and promoting homogeneous mixing at the molecular level. Subsequently, ethylene glycol is introduced, initiating a condensation reaction with citric acid to form a three-dimensional polyester network. This polymeric gel entraps the metal ions uniformly and ensures their intimate dispersion throughout the matrix, as shown in Scheme 2.

**Scheme 3:**
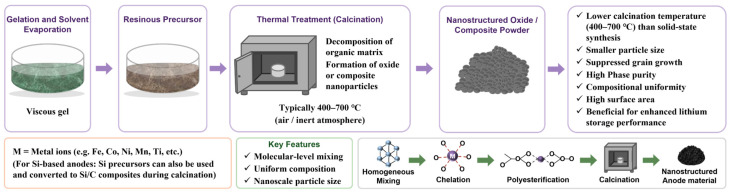
Schematic illustration of gelation and calcination steps of the Pechini method.

Upon gelation and solvent evaporation, the resinous precursor undergoes thermal treatment (calcination). During this stage, the organic matrix is decomposed and removed, while the metal ions react to form nanosized oxides or composite materials. The calcination temperature is typically much lower (400–700 °C) than that required for traditional solid-state synthesis, which helps maintain smaller particle sizes and suppress undesired grain growth [37,53]. The resulting powders often exhibit high phase purity, compositional uniformity, and surface area—all critical parameters for optimizing lithium storage behavior in anode materials, as described in Scheme 3 [13].

One of the most significant advantages of the Pechini process is its exceptional control over stoichiometry, which is particularly important for multicomponent oxides. Unlike traditional co-precipitation or solid-state methods, which often lead to phase segregation and inhomogeneity, the Pechini method ensures atomic-level mixing. This makes it especially suitable for synthesizing doped or complex anode materials such as Li_4_Ti_5_O_12_, Fe-doped TiO_2_, NiCo_2_O_4_, and Mn-based composite oxides [54,55,56,57]. The Li_4_Ti_5_O_12_ (LTO) is a spinel-type anode usually renowned for its outstanding cycling stability and zero-strain property [54,58]. The homogeneous distribution of Ti^4+^ and Li^+^ ions via the Pechini route enhances electrochemical uniformity, thereby improving capacity retention and rate capability. Similarly, transition metal oxide composites, such as MnFe_2_O_4_, NiCo_2_O_4,_ and Co_3_O_4_, benefit from the fine particle size and intimate mixing achieved by this method, leading to improved conductivity, stable solid–electrolyte interphase (SEI) formation, and reduced polarization during cycling [56,59,60]. The ability to tailor the composition by adjusting the metal-to-citric acid ratio and polymerization conditions offers tremendous flexibility for tuning structural and electrochemical properties. Furthermore, the Pechini method is scalable, cost-effective, and compatible with a wide range of metal precursors [61]. Its simplicity and reproducibility make it an attractive technique not only for academic research but also for potential industrial implementation in the synthesis of next-generation LIB anode materials.

### 2.3. Chelate Gel (Citric–Nitrate) Method

**Scheme 4:**
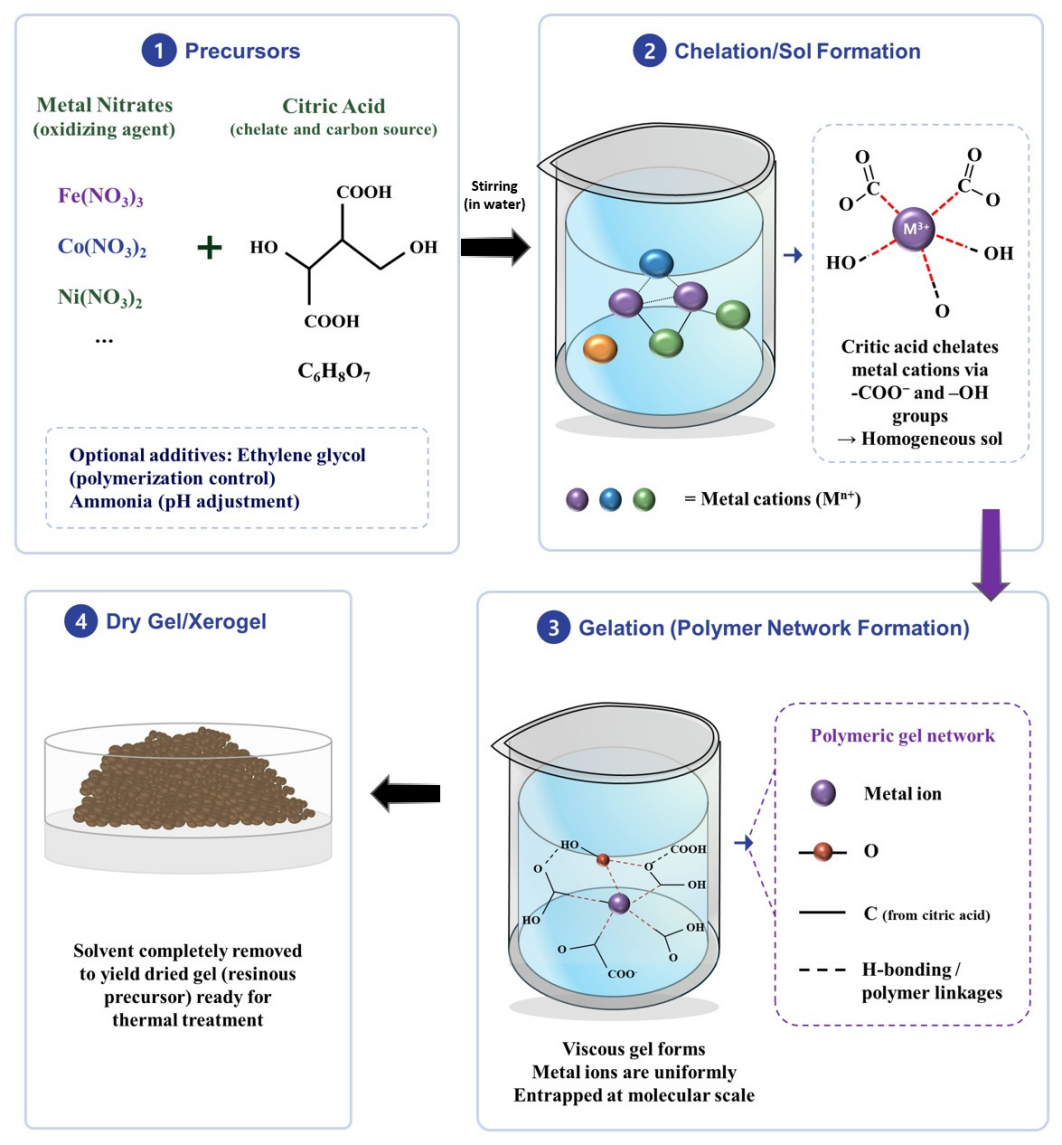
Schematic representation of precursors to gel formation steps of the chelate gel synthesis method.

**Scheme 5:**
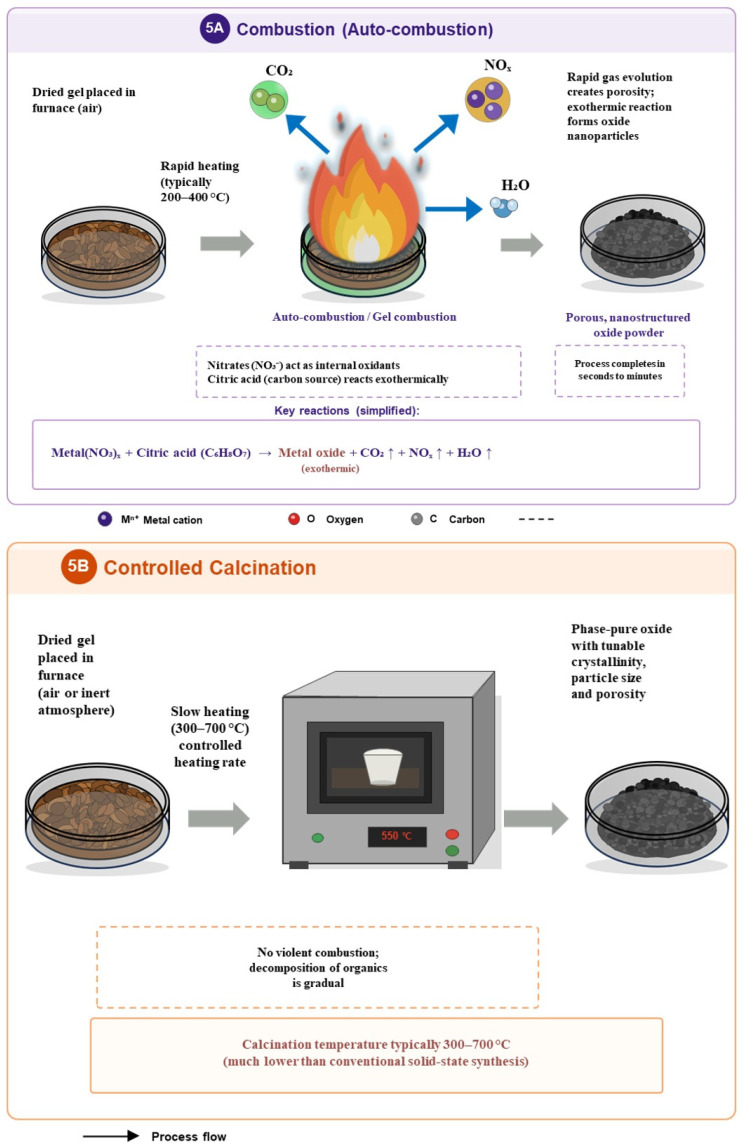
A schematic depiction of the different calcination routes: (**A**) Auto-combustion, and (**B**) Controlled calcination in the chelate gel synthesis method.

The citric–nitrate method, often referred to as chelate gel or combustion synthesis, is a widely used route for preparing porous, nanostructured transition-metal oxides. This method utilizes citric acid (C_6_H_8_O_7_) as a multifunctional agent—serving both as a chelating ligand and a carbon source—in combination with nitrate salts (typically metal nitrates such as Fe(NO_3_)_3_, Co(NO_3_)_2_, Ni(NO_3_)_2_, etc.) that act as oxidizing agents [27,62]. The chelation process begins with mixing aqueous solutions of metal nitrates and citric acid in stoichiometric ratios, often with additional agents such as ethylene glycol or ammonia to fine-tune pH and polymerization behavior. The carboxyl and hydroxyl groups of citric acid complex with the metal cations, forming a homogeneous sol. Upon mild heating (typically 80–120 °C), water gradually evaporates, and a viscous gel network forms, entrapping the metal ions uniformly at the molecular scale, as shown in Scheme 4. Sometimes, urea or glycine is incorporated as a fuel source [63]. This gel, when subjected to thermal treatment, undergoes auto-combustion or controlled calcination, depending on the desired material characteristics. During combustion, the nitrates serve as internal oxidants, reacting exothermically with the carbon-rich organic matrix of citric acid. This results in a self-propagating combustion reaction that rapidly decomposes the organic components, releasing gases such as CO_2_, NO_x_, and H_2_O, and simultaneously forming oxide nanoparticles, as presented in Scheme 5 [64,65]. The rapid gas evolution promotes porosity, while the exothermic nature of the reaction accelerates the synthesis, often completing in a matter of minutes. This “gel combustion” route therefore eliminates the need for prolonged high-temperature sintering, making it attractive for large-scale, energy-efficient production. Alternatively, slow calcination of the gel under air or inert gas can also yield phase-pure oxides with tunable crystallinity and particle size, as shown in Scheme 5.

One of the primary benefits of the citric–nitrate gel method lies in its simplicity and low cost. It employs inexpensive, water-soluble reagents and can be conducted at ambient pressure without specialized reactors or templates. Moreover, the molecular-level mixing of metal ions and organic ligands ensures excellent compositional homogeneity, a critical factor in multicomponent oxides such as Li_4_Ti_5_O_12_, LiMn_2_O_4_, or doped variants of LiFePO_4_ [63,66,67]. The method is also particularly well-suited for synthesizing porous and nanocrystalline materials, owing to the high gas evolution and short reaction duration during combustion. These nanostructured morphologies offer high surface area, improved electrolyte contact, and short lithium–ion diffusion paths—attributes that directly enhance electrochemical performance when such materials are used as electrodes in lithium–ion batteries [68]. In addition, the chelate gel (combustion) method is highly adaptable to a wide range of metal cation combinations and can be tuned by altering the citric acid-to-nitrate ratio, pH, and heating rates. For example, a higher citric–to-nitrate ratio can promote carbonaceous residue formation and influence combustion behavior, while variations in pH affect the gelation kinetics and polymer cross-linking. This tunability allows researchers to tailor porosity, particle size, and crystallinity, offering pathways to optimize electrode materials for specific battery chemistries [69,70,71]. The technique has been extensively applied to the synthesis of cathode materials: LiMn_2_O_4_, LiNi_0.5_Mn_1.5_O_4_, LiCoO_2_, and even micro/nanostructured SiO_x_/C composites for high-capacity anodes [72,73,74,75]. When combined with conductive additives or polymer binders, gel-derived oxide or hybrid structures could display improved cycling stability, rate performance, and structural integrity during repeated lithiation/delithiation cycles. As such, the citric–nitrate method stands out as a versatile, scalable, and compositionally controllable synthesis route for next-generation anode and cathode materials in lithium–ion battery systems.

## 3. Gel-Assisted Synthesis of Anode Materials

Gel-based methods, including sol-gel, polymeric gel, and chelate gel routes, offer a scalable approach to synthesizing micro and nanostructured composite anodes with controlled composition and porosity. Chelating agents enable uniform precursor distribution, leading to fine, porous structures after thermal treatment that enhance lithium–ion transport. This approach has been successfully applied to transition-metal oxides and silicon-based composite anodes, resulting in enhanced capacity, rate performance, and cycling stability.

### 3.1. Silicon-Based Composites

**Figure 1:**
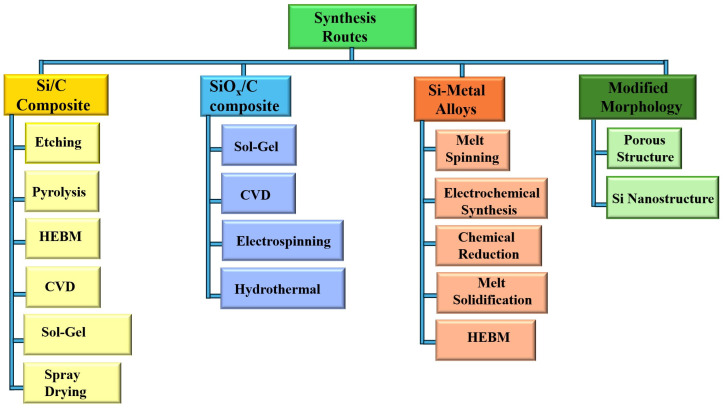
Common synthesis routes for Si-based anode materials.

Silicon is a promising anode material for lithium–ion batteries due to its exceptionally high theoretical capacity (~4200 mAh g^−1^); however, its practical application is hindered by severe volume expansion (>300%) during lithiation, leading to mechanical degradation and poor cycle stability [76,77]. Various synthesis methods aim to address these challenges, including porous architecture, nanostructuring, alloying with other elements, and carbon coating to improve mechanical stability and mitigate volume expansion. An overview of the various methods for synthesizing Si-based anode materials, compiled from several published review articles, is shown in Figure 1 [14,15,17,18].

**Figure 2:**
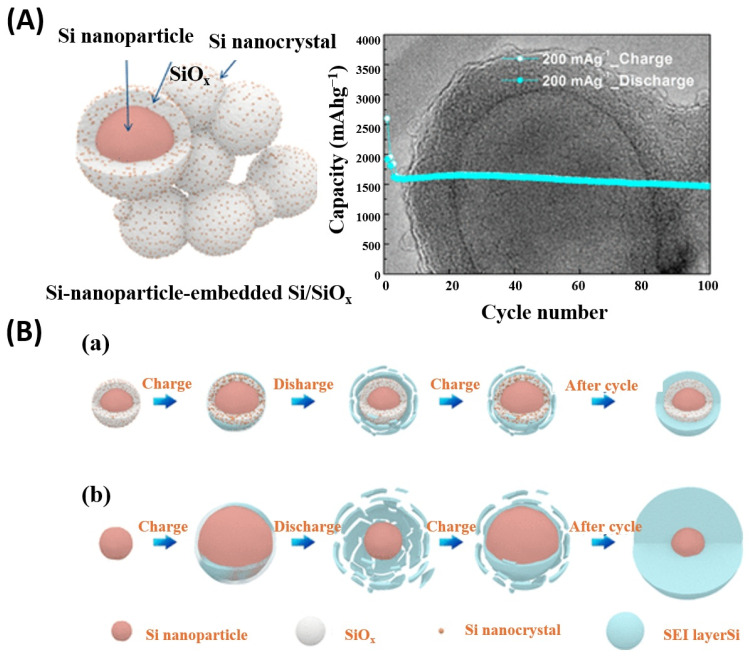
(**A**) Schematic illustration and HRTEM image of the C-coated Si-nanocrystal-embedded SiO_x_ nanocomposite. (**B**) Schematic illustration of SEI formation on the dual-size C-coated Si-nanocrystal-embedded SiO_x_ nanocomposite (**a**), and bare Si nanoparticles (**b**) during lithiation and delithiation. Adapted with permission [80], Copyright © 2015 American Chemical Society.

Gel-precursor-based sol–gel synthesis routes offer a compelling strategy to address these challenges by enabling the formation of nanostructured, porous silicon composites embedded in carbonaceous matrices, with or without metal oxides [78,79]. The electrochemical performance of silicon-based anodes is closely tied to the morphology produced by gel precursor chemistry. Factors like particle size, porosity, carbon distribution, conductive network connectivity, and structural flexibility play key roles in determining lithium diffusion behavior, electron transport kinetics, SEI stability, and mechanical durability. Gel-derived approaches offer distinct benefits by allowing the simultaneous optimization of these structural features across multiple length scales. The morphology of silicon-based anodes produced via polymeric and chelate gel precursors is largely determined by precursor chemistry, raw material selection, gelation kinetics, and thermal processing conditions. Unlike traditional solid-state synthesis, gel-based approaches enable molecular-level blending of silicon sources, carbon precursors, and functional additives, offering precise control over particle size, pore structure, and the formation of a conductive network. This structural control is essential for silicon-based anodes, as their electrochemical degradation is primarily driven by significant volume changes, particle fragmentation, and an unstable solid–electrolyte interphase (SEI) during repeated lithiation/delithiation cycles [10]. For example, the sol–gel reaction of triethoxysilane with commercially available Si nanoparticles produces a dual-size silicon nanocrystal-embedded SiO_x_ nanocomposite that serves as a high-capacity lithium storage material and promotes the formation of a thin and stable SEI film to withstand repeated volume expansion and contraction (Figure 2) [80].

One of the most influential parameters in gel-derived synthesis is the choice of silicon precursor. Silicon alkoxides such as tetraethyl orthosilicate (TEOS) and tetramethyl orthosilicate (TMOS) are commonly used due to their high reactivity in hydrolysis and condensation reactions [81,82,83]. Typically, silicon alkoxides (e.g., TEOS or TMOS) or silicon nanoparticles are dispersed within an organic gel network, often formed by chelating agents like citric acid or polyols, and then thermally treated to produce Si/C or silicon oxide/C hybrids with controlled porosity and nanoscale confinement. These gel-derived Si/C or SiO_x_/C anodes exhibit improved structural integrity, enhanced electronic conductivity, and better accommodation of volumetric changes, resulting in significantly improved cycling performance. During gel formation, hydrolyzed silanol groups undergo polycondensation to form interconnected Si–O–Si networks. The hydrolysis rate depends on water content, catalyst type, solvent polarity, and pH. Under acidic conditions, hydrolysis is slower, producing uniform linear polymer structures with smaller pores. In contrast, alkaline conditions accelerate condensation, leading to highly branched clusters with larger pore channels and rougher morphologies. Therefore, pH control is essential for tailoring silicon nanostructures and porosity [81,84,85]. The raw materials used as chelating agents and polymerization media significantly impact the final morphology. Citric acid is one of the most commonly used chelating ligands due to its carboxyl and hydroxyl groups, which allow strong coordination with metal or silicon-containing species [86]. During polymeric gel synthesis, citric acid forms stable complexes that prevent premature precipitation and maintain molecular-level homogeneity. Ethylene glycol is often added to facilitate polyesterification reactions and form three-dimensional polymer networks [87,88]. The ratio of chelating ligands to ethylene glycol influences gel viscosity, cross-link density, and carbon yield during calcination [89]. A higher citric acid content typically increases the residual carbon after pyrolysis, resulting in thicker carbon coatings and improved electrical conductivity. However, excessive organic content can cause structural collapse or the formation of dense carbon agglomerates, thereby limiting access to the active surface. In polymeric gel routes, the choice of solvent greatly affects drying behavior and pore development. Polar solvents such as ethanol and water promote homogeneous dissolution of precursors, while mixed-solvent systems can control evaporation rates and gel shrinkage [81,83]. Slow solvent evaporation typically yields crack-free monolithic gels with uniform mesoporosity, whereas rapid evaporation can induce capillary stress and structural damage. To preserve porous frameworks and prevent pore collapse, freeze-drying methods are commonly used [90]. In graphene-aerogel-supported SiOC systems, freeze-drying helps retain three-dimensional conductive networks and interconnected macropores, thereby enhancing electrolyte penetration and electron transport pathways [91].

**Figure 3:**
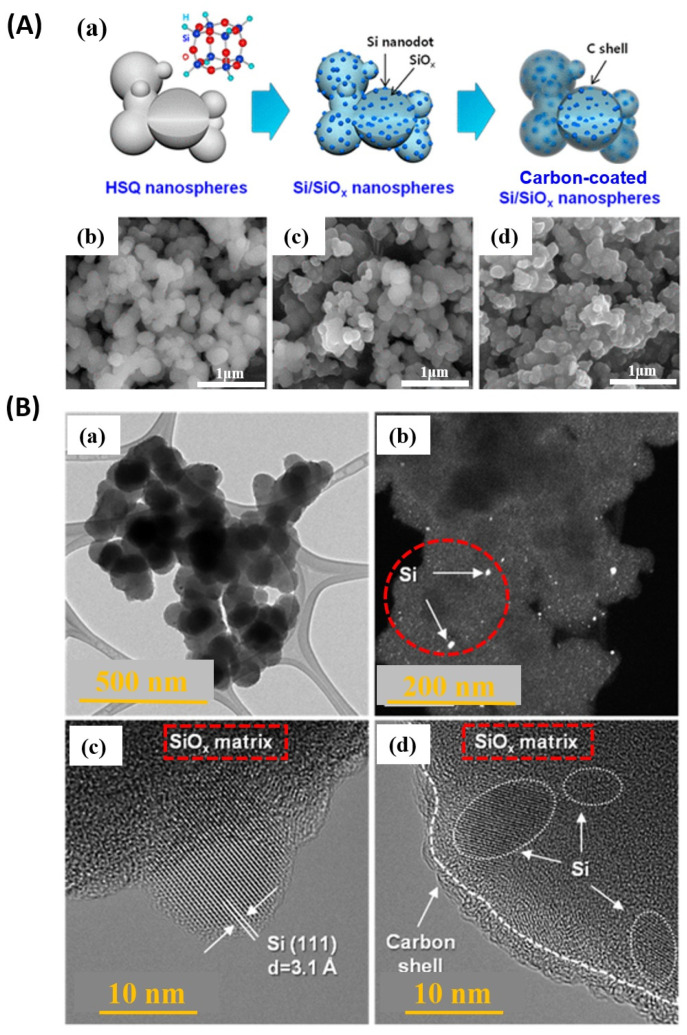
(**A**) Schematic representation for the C-coated Si/SiO_x_ nanospheres synthesis (**a**). FESEM images of HSQ nanospheres prepared by a sol−gel reaction of triethoxysilane (**b**), HSQ-derived Si/SiO_x_ nanospheres after heat treatment (**c**), and C-coated Si/SiO_x_ nanospheres after the pyrolysis of pitch (**d**). (**B**) HSQ-derived Si/SiO_x_ nanospheres. (**a**) Bright-field TEM image and (**b**) dark-field TEM image; HRTEM image (**c**) a crystalline Si nanodot embedded in the HSQ-derived Si/SiO_x_ nanospheres, and (**d**) C-coated Si/SiO_x_ nanospheres. Adapted with permission [92], Copyright © 2014 American Chemical Society.

Another major advantage of gel-derived synthesis is the precise control over nanostructure morphology. Gel precursor chemistry enables nanoscale confinement of silicon domains within conductive matrices, as shown in Figure 3 [92]. Nanostructured silicon domains synthesized through polymeric gel routes experience lower absolute strain because smaller particles distribute stress more uniformly. A smaller particle size also prevents crack formation and mechanical breakdown. As a result, nanosilicon composites prepared by gel methods typically exhibit better cycling stability than bulk silicon electrodes. In many gel-derived Si/C or Si/SiO_x_/C systems, silicon nanoparticles or silicon-containing phases are evenly dispersed within the conductive rigid frameworks formed during polymer pyrolysis [93,94]. The uniform distribution of precursors minimizes large-scale phase segregation and enables the formation of nanoscale silicon domains within conductive carbon matrices, thereby effectively reducing mechanical stress buildup during lithiation and delithiation [91,95,96]. It also prevents particle agglomeration and avoids localized stress buildup during cycling. Additionally, nanoconfined silicon domains lower strain gradients and reduce lithium diffusion distances, thereby enhancing electrochemical kinetics.

**Figure 4:**
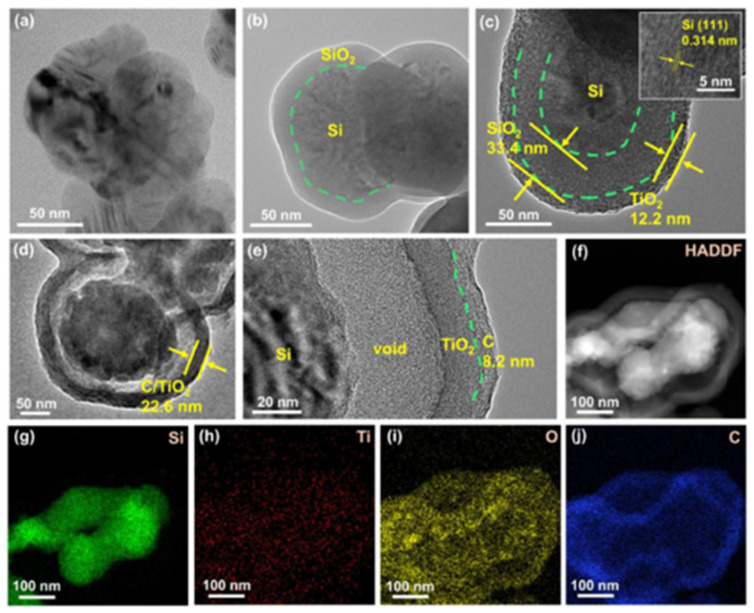
TEM images of (**a**) Si, (**b**) SiO_2_@Si, (**c**) TiO_2_@ SiO_2_@Si, (**d**) and (**e**) CTS; (**e**) HADDF-STEM image and (**f**–**j**) corresponding EDS element mapping of CTS; reproduced with permission [82], Elsevier.

Gel chemistry also enables the fabrication of porous and hollow structures that can effectively buffer silicon volume changes [97]. Z. Liu et al. presented yolk–shell-structured silicon suboxide (SiO_x_/C) microspheres as anodes, featuring a semi-graphitic carbon coating on both the outer and inner surfaces, prepared through a sequential process of the sol–gel method, selective etching, and chemical vapor deposition [98]. This distinctive yolk–shell structure effectively manages the inherent volume changes by providing larger inner voids for accommodation, achieving a high specific capacity (1165 mA h g^−1^), excellent rate capability (725 mA h g^−1^ at 1000 mA g^−1^), and outstanding cycling stability (972 mA h g^−1^ after 500 cycles at 500 mA g^−1^). K. Wang et al. developed a yolk–shell structure of carbon-TiO_2_-Si (CTS) via a template-based sol–gel process [82]. The Si nanoparticles are enclosed within double carbon/TiO_2_ shells, with ample void space between the Si core and the carbon/TiO_2_ shells (Figure 4). This yolk–shell design with concentric double shells significantly enhances the structural stability of the electrodes. With these techniques, precise control over the material composition and structure can be achieved, making it suitable for creating complex architecture.

**Figure 5:**
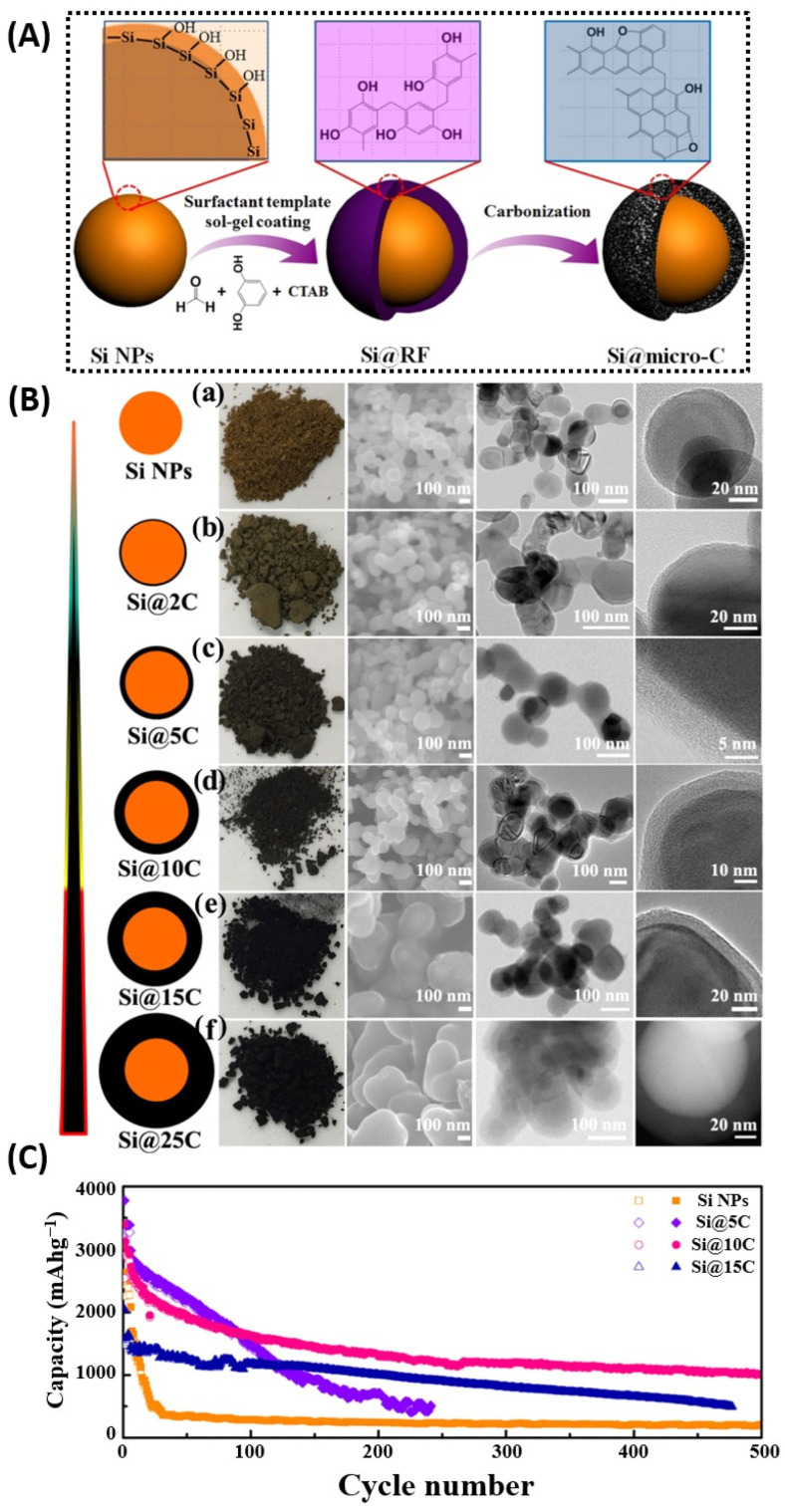
(**A**) Schematic illustration of how the surfactant template sol–gel approach synthesizes carbon interfacial layers on silicon nanoparticles. (**B**) Representing optical, SEM, and TEM images of carbon-coated silicon (Si@C) core–shell nanoparticles with controllable coating thickness of: (**a**) 0 nm, (**b**) 2 nm, (**c**) 5 nm, (**d**) 10 nm, (**e**) 15 nm, and (**f**) 25 nm, respectively. (**C**) Electrochemical cycling performance of Si@C core–shell nanoparticles with tuned layer thickness. Reproduced with permission [99], Elsevier.

The polymeric gel method is also highly effective for creating uniform carbon coatings with optimized thickness on silicon particles, as shown in Figure 5A [99]. The thickness of the carbon coating layer can be precisely adjusted by varying the amounts of silicon nanoparticles and resorcinol-formaldehyde (RF) organic polymers (Figure 5B). Silicon itself has inherently low electronic conductivity, which hinders charge-transfer kinetics and leads to polarization during cycling. During calcination, organic polymer chains carbonize, forming conductive amorphous carbon layers that enhance electronic conductivity and stabilize the electrode/electrolyte interface. This uniform carbon coating also reduces direct contact between silicon and the electrolyte, limiting excessive SEI growth and minimizing irreversible lithium consumption. These conductive carbon matrices generated during gel pyrolysis play a central role in electrochemical enhancement (Figure 5C). The formation of interconnected conductive networks around silicon particles using carbon generated from other precursors, such as citric acid, glucose, sucrose, or polymers, has also been widely reported [100,101,102,103,104]. These carbon frameworks enable continuous electron flow and lower internal resistance. In many gel-derived Si/C composites, carbon encapsulation also stabilizes the electrode/electrolyte interface by limiting direct contact between silicon and the electrolyte.

**Figure 6:**
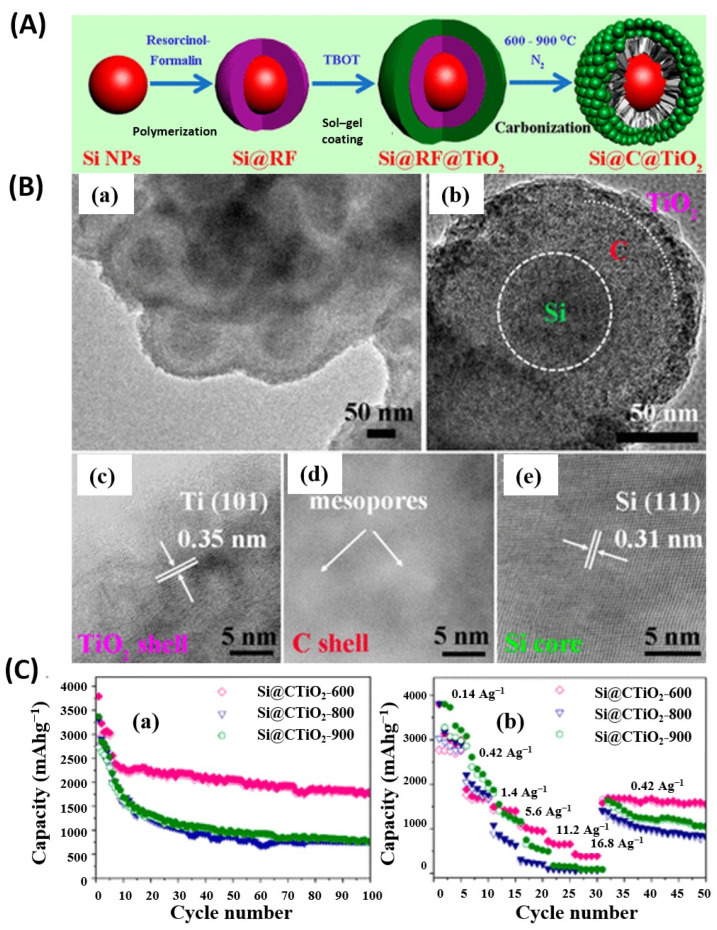
(**A**) Schematic depiction of the Si@C@TiO_2_ core–shell–shell nanoparticles anode synthesis steps via the two-step sol–gel coating process. (**B**) TEM images at various magnifications of the as-prepared Si@C@TiO_2_-600 core–shell nanoparticles. (**a**,**b**) HRTEM images of the (**c**) TiO_2_ shell, (**d**) Carbon shell, and (**e**) Si core. (**C**) Electrochemical cycling performances at a current density of 420 mA g^–1^ (**a**), and rate capability (**b**) of Si@C@TiO_2_-600, Si@C@TiO_2_-800, and Si@C@TiO_2_-900 core–shell–shell nanoparticle electrodes. The capacities were assessed based on Si weight. Adapted and redrawn with permission [105], Copyright © 2016 American Chemical Society.

The calcination atmosphere and temperature play a crucial role in shaping the final morphology [105]. In inert environments such as Ar or N_2_, organic gel networks pyrolyze to produce conductive amorphous carbon matrices. At lower calcination temperatures (~500–700 °C), amorphous or partially crystalline silicon oxycarbide (SiOC) phases form, featuring high defect density and flexible structures that better accommodate volume expansion during cycling [91,106]. Higher temperatures promote graphitization and electrical conductivity but may also lead to silicon crystallization, grain growth, and pore collapse [107]. Thus, thermal treatment must be carefully optimized to balance conductivity and structural stability. The heating rate during thermal decomposition also significantly affects pore formation and particle morphology. Slow heating allows gradual decomposition of organic components and controlled gas release, producing dense, uniform microstructures, whereas rapid heating causes explosive gas release from decomposing nitrates and organic ligands, resulting in highly porous foam-like structures [64]. In citric–nitrate combustion systems, the exothermic reaction between nitrate oxidizers and carbonaceous fuel generates rapid gas release (CO_2_, H_2_O, NOx), forming interconnected mesoporous and macroporous channels throughout the material [108]. These pore structures are particularly beneficial for silicon anodes, as they provide internal free volume to accommodate expansion during lithiation [97]. Porosity development in gel-derived silicon anodes is strongly influenced by precursor composition and carbonization behavior. Organic additives such as glucose, sucrose, tartaric acid, and polyvinyl alcohol act as both carbon precursors and pore-forming agents [101,102,103,104]. During pyrolysis, the decomposition of these organics forms mesoporous structures with high specific surface area. Tartaric acid-assisted sol–gel methods produce finer particles and more pronounced mesoporosity compared to glucose-based systems. Gels derived from carboxylic acids generally exhibit greater pore volume and improved electrolyte accessibility, owing to greater gas release during decomposition. Porous architectures produced by gel combustion and polymer pyrolysis, such as RF resin polymer, are particularly effective at accommodating volumetric expansion (Figure 6A,B) [105]. Mesopores and macropores provide internal free space that buffers silicon swelling during lithiation. These void structures prevent catastrophic electrode fracture and maintain electrical contact between active particles and conductive matrices. Furthermore, interconnected pore channels facilitate electrolyte infiltration and shorten lithium-ion diffusion pathways, thereby improving the cyclability and rate capability (Figure 6B). A three-dimensional (3D) porous Si produced from a silica composite gel demonstrates excellent cycling stability (~0.98 mAh cm^−2^ after 200 cycles) and good rate performance even without a carbon coating layer [106]. The electrochemical behavior of gel-derived silicon anodes is also highly dependent on pore-size distribution [107,108]. Mesopores improve electrolyte accessibility and ion-diffusion kinetics, whereas macropores accommodate mechanical expansion and reduce stress concentrations. Micropores may increase irreversible capacity due to excessive SEI formation arising from their large surface area. Therefore, hierarchical pore structures combining micro, meso, and macroporosity are generally considered optimal for balancing capacity, conductivity, and cycling stability [109,110]. Gel chemistry provides exceptional flexibility for engineering such hierarchical porosity through careful control of precursor composition and decomposition behavior.

Incorporating graphene and carbon nanotubes (CNTs) enhances both morphology and mechanical strength. In graphene-integrated gel systems, the polymer precursor penetrates the graphene scaffold, forming layered heterostructures after pyrolysis [111], A silicon/silicon oxide/carbon/graphene (Si/SiO_x_/C/Graphene) composite is deliberately designed by the X. Wang group, using freeze-drying strategies in which the resorcinol-formaldehyde resin (RF) serves as the carbon source [90]. Both amorphous SiO_x_ and RF-derived carbon-embedded Si nanodomains effectively mitigate the mechanical stresses induced by Si volume changes, stabilize the electrode structure, ensure a stable electrode/electrolyte interface, and prevent electrode material degradation during charge/discharge. These materials feature interconnected conductive pathways and hierarchical porosity across micro, meso, and macroscales. Similarly, CNT incorporation improves conductive connectivity and prevents structural collapse by serving as flexible reinforcing frameworks. The core−shell structured CNT/meso-SiO_2_ nanocomposite anode synthesized via the sol−gel method was reported by Y. Yang et al. [108]. This sol–gel approach creates a highly porous composite sponge electrode with three-dimensionally interconnected carbon-Si-CNT skeletons. In this hierarchical structure, the macropores within the sponge connect to the mesopores in the meso-Si layer, facilitating Li^+^ diffusion, while the underlying CNT networks provide conductive pathways for electron transport. Additionally, the outer carbon coating on the meso-Si helps buffer volume expansion and prevents material shedding. These hybrid morphologies are highly effective in preserving Si electrode integrity during repeated volume changes.

**Figure 7:**
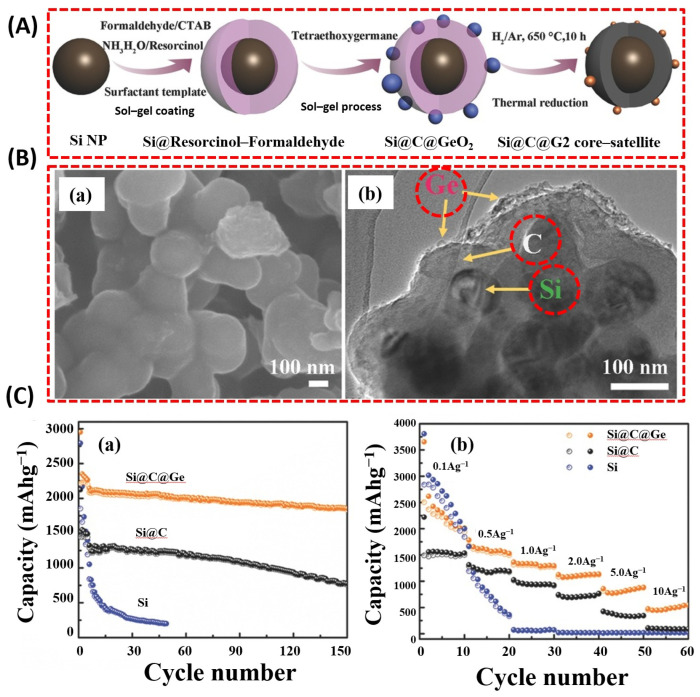
(**A**) Schematic depiction of the Si@C@Ge core–satellite nanoparticles synthesis steps. (**B**) SEM (**a**) and TEM (**b**) images showing Ge nanograins on Si@C core–shell nanoparticles (Si@C@Ge) morphology. (**C**) Electrochemical cycling performances at a current density of 500 mA g^−1^ (**a**), and rate capability (**b**) of Si, Si@C, and Si@C@Ge core–satellite nanoparticle electrodes. Adapted and redrawn with permission [113], Wiley and Sons.

Moreover, gel chemistry enables heteroatom doping (e.g., B, Ge, P) into the carbon coating layer, further enhancing the electrochemical behavior [112,113,114]. Heteroatom doping is another critical feature enabled by gel precursor chemistry. Nitrogen-containing polymers, urea, melamine, or ammonia can introduce nitrogen functionalities into carbon matrices during calcination. This doping enhances electronic conductivity, defect density, surface wettability, and lithium–ion adsorption and transport kinetics. Similarly, phosphorus doping alters the local electronic structure and improves interfacial stability [114]. The molecular-level mixing provided by gel-derived synthesis allows heteroatom dopants to be uniformly distributed throughout the matrix, resulting in consistent electrochemical activity. For example, W. Luo et al. introduced a novel heterostructured Si@C@Ge anode synthesized through a two-step sol–gel process (Figure 7A) [113]. The high-conductivity Ge nanograins on the surface enhance Li diffusion and electron transport, ensuring good ion accessibility. These Ge nanograins also act as a buffering cushion to mitigate mechanical strain on the electrode during lithiation/delithiation, providing excellent cycling stability over extended cycles, as shown in Figure 7B,C.

The morphology of gel-derived silicon composites is also strongly influenced by the silicon source. Nanosilicon powders tend to form more homogeneous composites due to their high surface area and ease of dispersion within gels, but they can also lead to excessive SEI formation because of the increased electrolyte contact area. In contrast, micron-sized silicon particles exhibit lower irreversible capacity loss but are more prone to internal stress and fracture during cycling [114]. To address these challenges, many studies use hierarchical micro and nanosilicon architectures or porous silicon [75,115]. These porous structures offer internal buffering spaces and enhanced mechanical stability. These approaches aim to balance the benefits of high surface area and reduced stress, enabling better cycle life and capacity retention. By optimizing silicon architecture, researchers can enhance lithium–ion battery performance while mitigating issues such as cracking and electrolyte degradation during repeated charge–discharge cycles [116]. It is important to note that both particle size and the volume fraction of Si active sites influence capacity. For instance, three Si/sol–gel/graphite anodes with varying Si contents were tested, and the anode with the lowest Si content showed the best capacity retention [117].

In addition to conventional xerogels, aerogel-based approaches have emerged as highly promising strategies for advanced silicon anodes due to their ultrahigh porosity, low density, and interconnected three-dimensional conductive frameworks. Silicon-containing aerogels are commonly synthesized through sol–gel reactions followed by supercritical drying or freeze-drying, which preserve the porous gel network by minimizing capillary collapse during solvent removal. Supercritical CO_2_ drying is particularly effective for maintaining nanoscale pore structures and generating highly open conductive architectures, whereas freeze-drying produces hierarchical macroporous frameworks through ice-template-assisted pore formation [118,119,120,121,122,123]. The resulting aerogel structures provide several electrochemical advantages for silicon anodes. First, the large pore volume and interconnected channels facilitate electrolyte penetration and shorten Li^+^ diffusion pathways. Second, the mechanically compliant porous framework can effectively buffer the severe volume expansion (>300%) of Si during lithiation/delithiation, thereby suppressing pulverization and electrode cracking. Third, aerogel-derived carbon or graphene networks significantly improve electrical conductivity and maintain structural integrity during long-term cycling. For example, Patil and Phadatare group reported carbon–silicon aerogels prepared via freeze-drying, in which the hierarchical porous architecture improved electrolyte accessibility and cycling stability [121,122]. Similarly, X. Hu et al. prepared a graphene/Si aerogel via freeze-drying and demonstrated enhanced cycling stability due to the flexible conductive network that accommodated Si volume changes [123]. When silicon-containing polymer precursors are incorporated into graphene aerogels, the resulting materials provide readily accessible electrolyte pathways and continuous conductive networks. These properties are particularly beneficial for high-rate lithium storage applications [119]. The rheological properties of the gel precursor also influence structural homogeneity. Highly viscous gels prevent sedimentation of silicon nanoparticles and promote uniform precursor distribution within the matrix. On the other hand, low-viscosity sols can lead to phase segregation or inconsistent particle dispersion. As a result, precursor concentration, polymerization time, and solvent evaporation rate must be carefully controlled to achieve homogeneous nanostructures.

**Figure 8:**
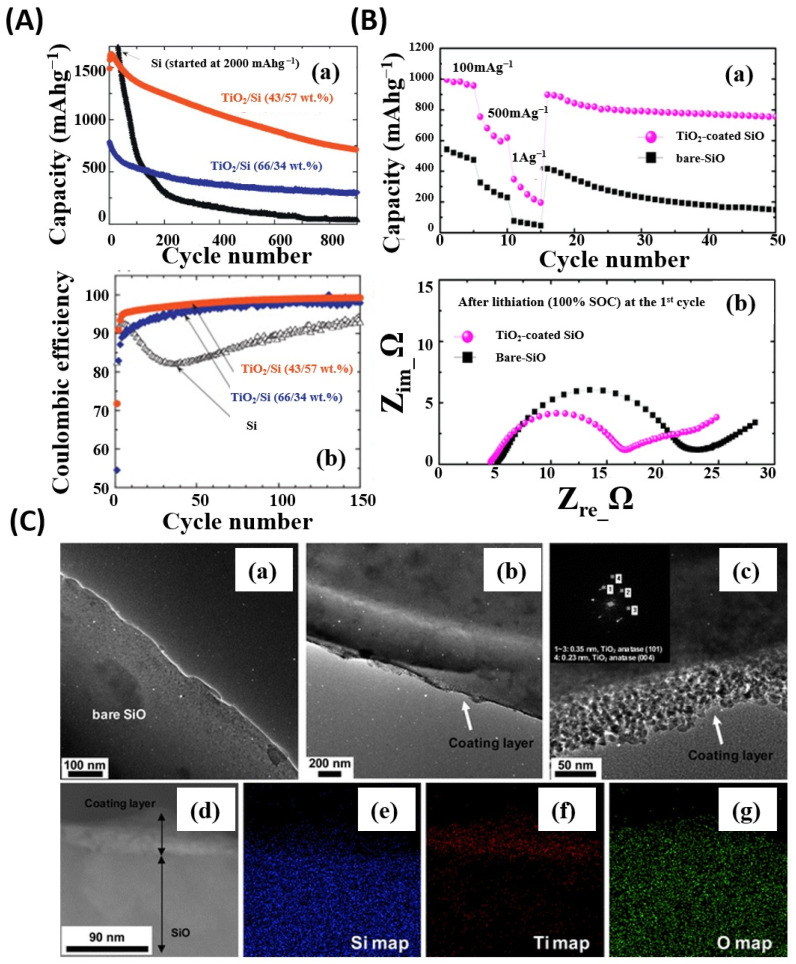
(**A**) Compares (**a**) the cycling performance, and (**b**) Coulombic efficiencies of Si and TiO_2_/Si composite electrodes; reproduced with permission [79], Elsevier. (**B**) Compares (**a**) the rate performance and (**b**) Nyquist plots of the bare SiO and TiO_2_-coated SiO. (**C**) TEM images of (**a**) bare SiO, and (**b**,**c**) TiO_2_-coated SiO. (**d**) STEM image and corresponding EDS elemental mappings of (**e**) Si, (**f**) Ti, and (**g**) O for the TiO_2_-coated SiO. Adapted and redrawn with permission [124], The Royal Society of Chemistry 2012.

**Figure 9:**
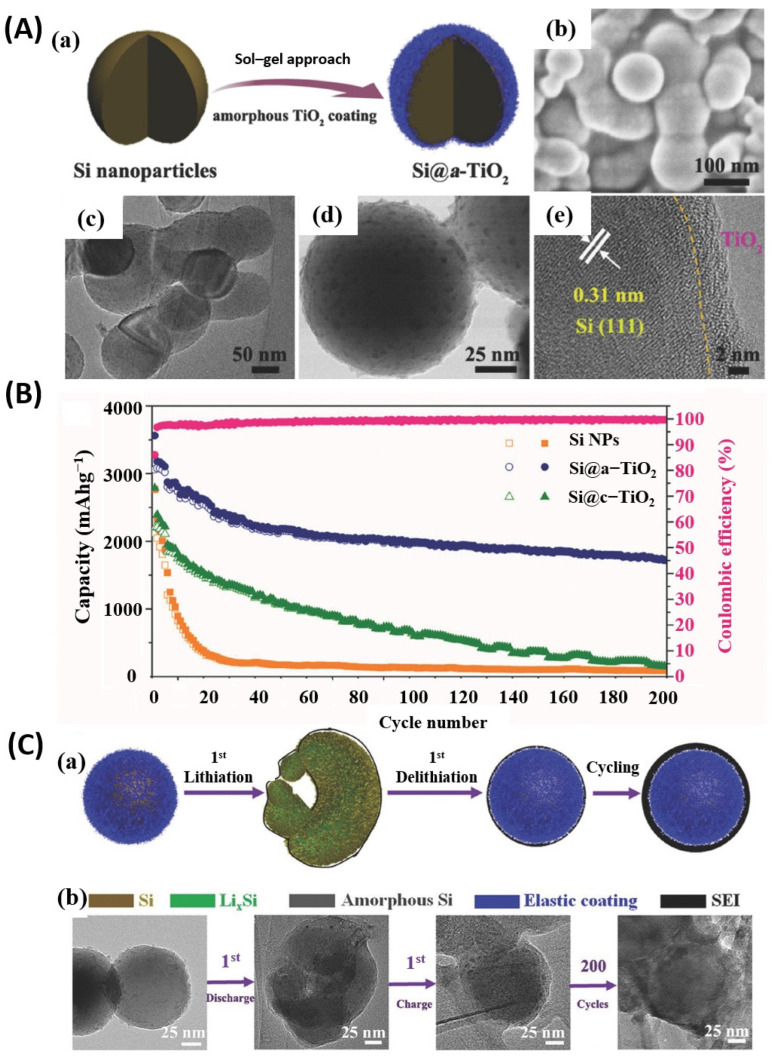
(**A**) Synthesis approach of the amorphous-TiO_2_-coated Si core–shell nanoparticles illustrated schematically (**a**). FESEM (**b**), TEM (**c**,**d**), and HR-TEM (**e**) images of Si@a-TiO_2_ nanoparticles. (**B**) Cycling performance comparison of the pristine Si, Si@a-TiO_2_, and Si@c-TiO_2_ nanoparticle electrodes at a current density of 420 mA g^−1^ (first activated with five cycles at 140 mA g^−1^). (**C**) Structural evolution of the amorphous-TiO_2_-coated commercial Si nanoparticle electrode during electrochemical cycling. Schematic sketch showing the structural maintenance of the Si@a-TiO_2_ nanoparticle electrode during lithium insertion and extraction (**a**), combined with the corresponding TEM images (**b**). Adapted with permission [128], Wiley and Sons.

Conformal metal oxide coatings can also be applied to silicon (Si) and silicon monoxide (SiO) anodes through a gel-precursor method. Typically, Si or SiO particles are dispersed in an alcohol-water solution containing metal alkoxide or inorganic salt precursors, such as titanium isopropoxide, aluminum isopropoxide, zirconium n-propoxide, or metal nitrates. Controlled hydrolysis and condensation reactions form a thin, uniform oxide layer (e.g., TiO_2_, Al_2_O_3_, ZrO_2_, or SiO_2_) on the particle surface, followed by mild calcination (300–700 °C) to crystallize or densify the coating. This approach ensures close interfacial contact and precise control over coating thickness, ranging from a few nanometers to tens of nanometers. For instance, H. Usui et al. prepared TiO_2_-coated Si composite by a simple sol–gel route using titanium isopropoxide, demonstrating that the composite effectively buffered volume expansion and suppressed continuous SEI growth, resulting in markedly improved cycling stability and coulombic efficiency, as presented in Figure 8A [79]. G. Jeong et al. used a simple sol–gel method with titanium isopropoxide to produce a TiO_2_-coated SiO anode, demonstrating that the nanoscale TiO_2_ shell effectively blocks exothermic reactions between lithiated SiO and the electrolyte solution, thereby improving conductivity and rate capability, as shown in Figure 8B,C [124]. Similarly, H. Zhu et al. reported an Al_2_O_3_ coating with a thickness of 10–15 nm applied to Graphite/silicon (G/Si) composites using a straightforward sol–gel method followed by high-temperature annealing [125]. W. Deng et al. produced SiO_x_@ZrO_2_@C Nanospheres via hydrolysis of zirconium and silicon alkoxides, in which the robust oxide coating enhanced structural integrity and maintained electrical contact during repeated lithiation/delithiation, thereby improving capacity retention [126]. In another example, D. Choi et al. used a sol–gel method to create ZrO_2_-coated SiO_2_ nanostructure, demonstrating that the chemically stable ZrO_2_ layer reduced interfacial impedance and improved long-term cycling performance [127]. These oxide shells not only provide mechanical buffering but also act as artificial interphases to regulate electrolyte decomposition, minimize irreversible lithium consumption, and promote stable SEI formation. The sol–gel method is also flexible enough to create multicomponent oxide-coated advanced core–shell and hybrid Si-based anodes. J. Yang et al. described Si nanoparticles coated with amorphous titanium oxide (TiO_2_) in a core–shell structure, which exhibit significantly improved electrochemical performance and high-safety lithium storage [128]. The amorphous TiO_2_ shells demonstrate elastic properties and provide excellent buffering capabilities (Figure 9). This buffering property helps to accommodate volume changes during charge and discharge cycles, preventing structural degradation of the Si nanoparticles. Consequently, the core–shell design enhances cycle life and stability, making it a promising material for next-generation lithium–ion batteries with improved safety and energy storage efficiency. A summary of the electrochemical performances and morphologies of various Si-based anode materials synthesized by sol–gel or modified sol–gel methods is presented in Table 1. It shows that the synthesis method, precursor, and chelating agents have a significant impact on morphology and electrochemical performance metrics.

### 3.2. Transition Metal Oxides-Based Anodes

Transition-metal oxides (TMOs) have been extensively investigated as alternative anode materials for lithium–ion batteries because many of them deliver theoretical capacities substantially higher than that of graphite [129,130]. Unlike graphite, which stores Li^+^ mainly through intercalation, most binary TMOs, such as Fe_2_O_3_, Fe_3_O_4_, Co_3_O_4_, NiO, CuO, and MnO_x_, store lithium through conversion reactions that involve the reversible reduction of metal oxides to metallic nanoparticles embedded in a Li_2_O matrix [129]. Despite their high theoretical capacity, TMO anodes generally suffer from intrinsically low electronic conductivity, large polarization, volume variation during conversion and reconversion, aggregation of metallic nanoparticles, unstable solid–electrolyte interphase formation, and low initial Coulombic efficiency [130,131]. These limitations strongly depend on particle size, crystallinity, porosity, carbon distribution, and the homogeneity of multi-cation oxide phases. Therefore, synthesis strategies capable of controlling composition, morphology, and nanoscale architecture are essential for improving the practical electrochemical performance of TMO anodes [130,132].

Among various wet-chemical methods, sol–gel synthesis is particularly attractive because it enables molecular and near-molecular level mixing of metal precursors, relatively low-temperature oxide formation, and tunable particle morphology [133]. In polymeric or chelate-gel routes, the final morphology of gel-derived TMO anodes begins with the choice of metal precursors. Metal nitrates [e.g., Fe(NO_3_)_3_·9H_2_O, Ni(NO_3_)_2_·6H_2_O, Co(NO_3_)_2_·6H_2_O, Mn(NO_3_)_2_·xH_2_O] are widely used because of their excellent water solubility and ability to act as oxidizing agents during combustion [134]. Metal acetates and chlorides are used when a slower decomposition or alternative complexation behavior is needed [13]. These metal salts are dissolved and complexed with organic chelating agents like citric acid, malic acid, ethylenediaminetetraacetic acid (EDTA), tartaric acid, glycine, glucose, or sucrose, and then undergo polyesterification with ethylene glycol or glycerol to create a cross-linked resin. The chelating agent coordinates metal ions and prevents premature precipitation, while subsequent drying and thermal decomposition of the gel yield nanosized oxide particles [135]. The ratios of citric acid to metal and citric acid to ethylene glycol are crucial in determining the gel structure, carbon yield, and pore formation. The type of organic additive significantly affects crystallite size and texture. Carboxylic acids like tartaric acid and malic acid typically result in smaller particles and higher surface areas due to the large volume of gases released during their decomposition, which generates mesoporosity. On the other hand, glucose and sucrose tend to produce higher carbon contents and more uniform conductive coatings, though the materials may have lower surface areas [89,136,137]. The pH of the solution plays a key role in metal-ligand complexation and gelation kinetics. At low pH, protonated carboxyl groups bind metal ions more slowly, resulting in uniform gels and smaller oxide particles. Raising the pH (often with ammonia) promotes carboxyl group deprotonation, strengthens chelation, and speeds up polymerization. However, excessively high pH can lead to metal hydroxide precipitation, causing phase segregation and nonuniform morphology [40,138]. Therefore, precise pH control is crucial to maintain compositional homogeneity and prevent impurity formation. Slow drying produces dense xerogels, whereas rapid evaporation or microwave-assisted heating can create foam-like structures with abundant pores. Freeze-drying is particularly effective for preserving three-dimensional porous networks by minimizing capillary stresses that otherwise collapse the gel skeleton [64,91,139]. Such highly porous aerogel-like structures are advantageous for TMO anodes because they improve electrolyte infiltration and shorten lithium diffusion paths. In citric–nitrate methods, the ratio of citric acid (fuel) to nitrate ions (oxidizer) determines the combustion intensity and porosity. Fuel-rich mixtures result in strong exothermic reactions and rapid gas release (CO_2_, NO_3_, H_2_O), creating highly porous foam-like structures. Oxidizer-rich systems produce gentler combustion and denser particles. It is crucial to optimize this ratio, as excessive combustion can cause local overheating and grain coarsening, while insufficient fuel may result in incomplete decomposition and leftover carbonaceous impurities [27,70,140].

For binary conversion-type oxides, sol–gel synthesis mainly improves performance by reducing particle size, shortening Li^+^ diffusion pathways, and creating porous or loosely packed structures that can accommodate volume changes [137]. Co_3_O_4_ is a representative example. It can theoretically store eight Li^+^ per formula unit, corresponding to a capacity of about 890 mAh g^−1^, but practical performance is limited by poor conductivity and conversion-induced mechanical degradation [141]. Iron-based oxides are also widely studied because of their low cost, natural abundance, and high theoretical capacity. Fe_2_O_3_ and Fe_3_O_4_ undergo conversion to metallic Fe and Li_2_O during lithiation. However, like Co_3_O_4_, their practical capacity retention is restricted by poor conductivity and structural pulverization. Sol–gel-derived iron oxide nanoparticles can partially address these issues by forming smaller crystallites and porous aggregates. In mixed ferrite systems, the advantage of sol–gel synthesis becomes even more pronounced.

**Figure 10:**
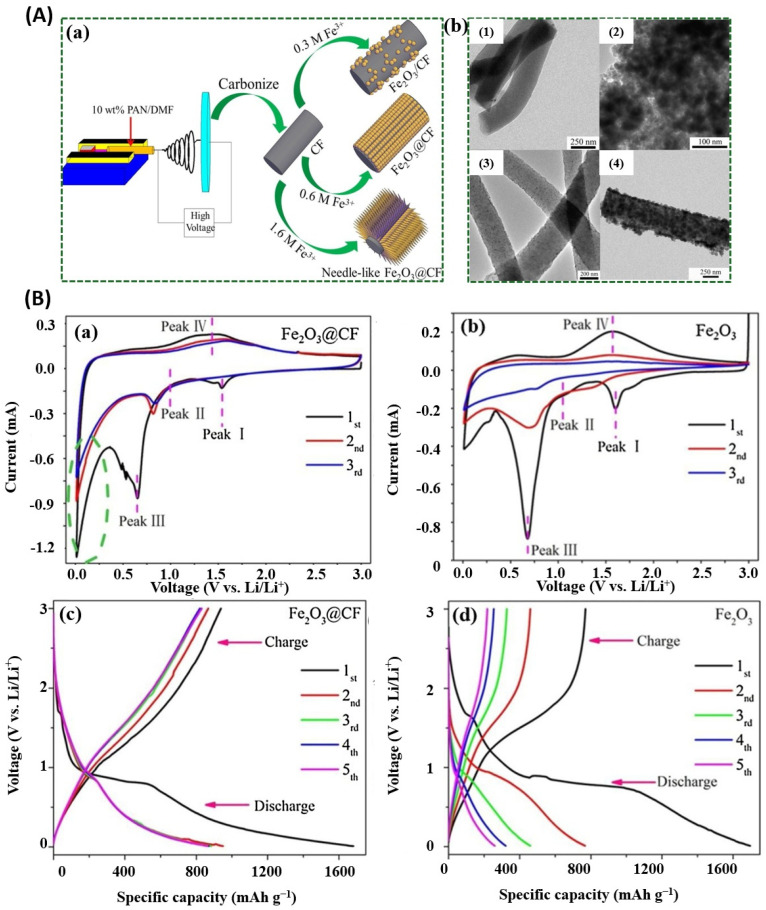
(**A**) Synthesis and morphology of Fe_2_O_3_-carbon fiber composite anodes: (**a**) a schematic representation of the process for fabricating Fe_2_O_3_-carbon fiber composites through electrospinning and carbonization, followed by sol–gel growth of Fe_2_O_3_ using varying Fe^3+^ concentrations, (**b**) TEM images of (1) carbon fibers, (2) Fe_2_O_3_ nanoparticles, (3) ultrafine Fe_2_O_3_/CF, and (4) Fe_2_O_3_@CF composites. (**B**) Electrochemical behavior of Fe_2_O_3_–carbon fiber composite anodes: CV curves for (**a**) Fe_2_O_3_@CF and (**b**) bare Fe_2_O_3_, and charge–discharge profiles of (**c**) Fe_2_O_3_@CF and (**d**) bare Fe_2_O_3_. The Fe_2_O_3_@CF composite shows improved electrochemical reversibility owing to uniform Fe_2_O_3_ loading on the conductive carbon-fiber scaffold. Adapted from [142] with permission, Elsevier.

Fe_2_O_3_-based conversion anodes are promising because of their high theoretical capacity, low cost, and environmental compatibility, but their practical performance is limited by poor conductivity and large conversion-induced volume changes [142]. As shown in Figure 10(Aa), a combined electrospinning and sol–gel strategy was used to grow Fe_2_O_3_ directly on one-dimensional carbon fibers, where the Fe^3+^ precursor concentration controlled the final morphology from Fe_2_O_3_/CF to Fe_2_O_3_@CF and needle-like Fe_2_O_3_@CF structures. The TEM images in Figure 10(Ab) show that Fe_2_O_3_ nanoparticles were uniformly anchored on the carbon fiber surface, reducing particle aggregation and forming mesoporous contact between Fe_2_O_3_ and the conductive carbon scaffold. This architecture improves electrolyte accessibility, shortens Li^+^ diffusion pathways, and provides continuous electron transport. The CV and charge–discharge profiles in Figure 10(Ba–Bd) further demonstrate that Fe_2_O_3_@CF exhibits more stable redox behavior and better capacity retention than bare Fe_2_O_3_. The optimized Fe_2_O_3_@CF electrode delivered 634 mAh g^−1^ after 150 cycles at 50 mAh g^−1^, with lower charge–transfer resistance than pure Fe_2_O_3_, confirming that gel-assisted growth on carbon fibers is an effective strategy for improving the structural stability and electrochemical reversibility of Fe_2_O_3_-based anodes [142]. Carbon-coated Fe_2_O_3_ represents an effective sol–gel-assisted strategy to improve conductivity and cycling stability. In this work, a nanoporous Fe_2_O_3_ xerogel was first prepared by a simple sol–gel route and then converted into Fe_3_O_4_/C via carbon CVD, in which carbon deposition simultaneously reduced Fe_2_O_3_ to Fe_3_O_4_ and formed an amorphous carbon coating. The resulting Fe_2_O_3_/C composite consisted of nanosized Fe_2_O_3_ particles coated with a thin carbon layer, which helped suppress particle aggregation, buffer volume expansion, and maintain electronic contact during repeated lithiation and delithiation. As an anode, the Fe_2_O_3_/C electrode delivered a high reversible capacity of about 850 mAh g^−1^ at 0.1 A g^−1^ with stable cycling and good rate capability, retaining about 570 mAh g^−1^ even at 1 A g^−1^. This study highlights that combining sol–gel-derived oxide precursors with a carbon coating is a practical route to designing high-capacity, structurally stable Fe_2_O_3_-based anodes [143].

**Figure 11:**
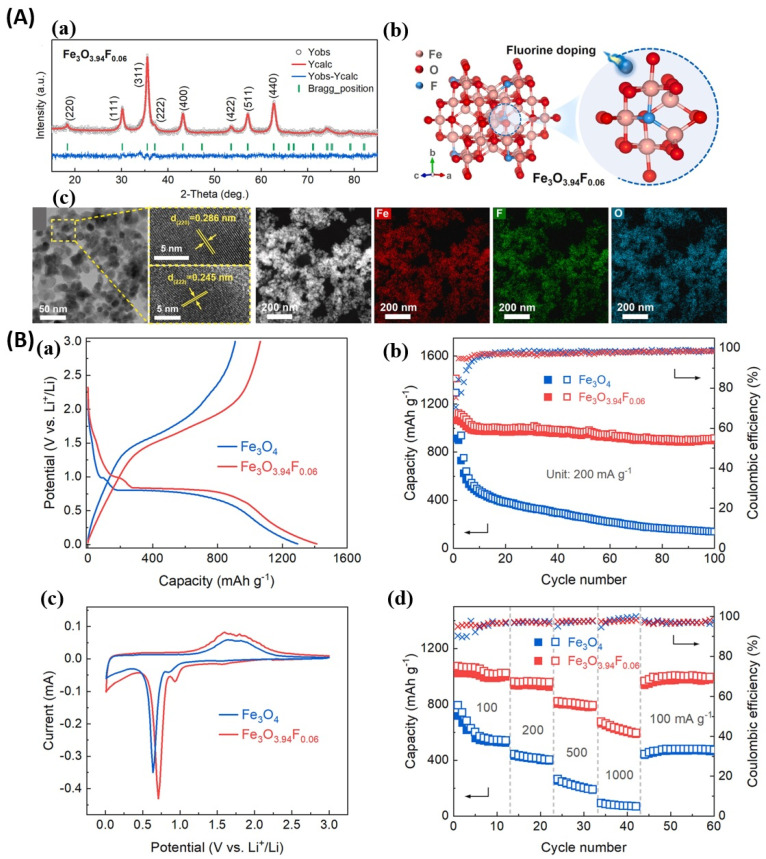
(**A**) Structural and morphological characterizations for Fe_3_O_3.94_F_0.06_: (**a**) XRD Rietveld refinement, (**b**) schematic illustration of F doping, (**c**) HR-TEM and EDS mappings of Fe_3_O_3.94_F_0.06_. (**B**) Comparison of electrochemical behavior of Fe_3_O_4_ and Fe_3_O_3.94_F_0.06_ anodes in LIBs: (**a**) initial charge-discharge curves at 200 mA g^−1^, (**b**) cycling performance, (**c**) CV scans at 0.1 mV s^−1^, (**d**) rate capability. Reproduced with permission [144], Elsevier.

Anion doping offers an intrinsic strategy to improve the sluggish Li^+^ kinetics of conversion-type Fe_3_O_4_ anodes by modifying the local bonding environment and electronic structure, rather than relying only on external carbon coating or morphology control. As shown in Figure 11(Aa), fluorine-doped Fe_3_O_4_, Fe_3_O_3.94_F_0.06_, was successfully prepared through a sol–gel process. The XRD refinement confirms that the doped sample retains the inverse-spinel Fe_3_O_4_ structure, while the TEM and elemental mapping results show nanosized particles with homogeneous Fe, O, and F distributions, indicating successful incorporation of F into the oxide framework. The schematic in Figure 11(Ab) illustrates that partial substitution of O_2_ by F modifies the Fe–O/F coordination environment, which can enlarge local diffusion sites for Li^+^ transport. This structural modification is reflected in electrochemical behavior: Figure 11(Ba,c) shows that Fe_3_O_3.94_F_0.06_ exhibits lower polarization and more reversible redox behavior than pristine Fe_3_O_4_ during lithiation and delithiation. The cycling data in Figure 11(Bb) further demonstrate the stabilizing effect of F doping, with Fe_3_O_3.94_F_0.06_ retaining 902.3 mAh g^−1^ after 100 cycles at 200 mA g^−1^, whereas undoped Fe_3_O_4_ decays sharply to 137.2 mAh g^−1^. In addition, the rate performance in Figure 11(Bd) confirms faster reaction kinetics, as the doped electrode delivers 596.8 mAh g^−1^ even at 1000 mA g^−1^ and recovers 1005.8 mAh g-1 when the current density returns to 100 mA g^−1^. These results indicate that sol–gel enabled fluorine doping effectively enhances both structural reversibility and charge-transfer/Li^+^-transport kinetics in Fe_3_O_4_ based conversion anodes [144].

Mixed transition-metal oxides, including spinel ferrites and manganates, are especially suitable for polymeric or chelate–gel synthesis because their electrochemical behavior depends strongly on cation distribution and phase purity. Compared with binary oxides, mixed oxides can provide multiple redox centers, improved structural robustness, and, in some cases, combined conversion/alloying reactions. ZnFe_2_O_4_ is a typical example, where Fe participates in conversion reactions, while Zn can contribute to additional alloying/dealloying with Li. However, the large volume expansion and poor conductivity of ZnFe_2_O_4_ remain major challenges. In situ carbon-coated ZnFe_2_O_4_ prepared by a sol–gel method has been reported to improve electron transport and mitigate volume expansion, delivering high reversible capacity and better rate behavior than bare ZnFe_2_O_4_ [145]. Manganese-based mixed oxides such as ZnMn_2_O_4_ and MgMn_2_O_4_ have also been prepared by citric acid, sucrose, or other chelator-assisted sol–gel routes. These materials are attractive because Mn is more abundant and less toxic than Co or Ni, while the spinel framework can provide structural tolerance during repeated lithiation and delithiation. Cai et al. [146] synthesized block-like ZnMn_2_O_4_ using a citric acid sol–gel combustion method and emphasized the importance of xerogel decomposition behavior in determining the final morphology. Similarly, MgMn_2_O_4_ nanoparticles synthesized using different chelators and solvents showed that chelator and solvent selection directly influenced particle size, cationic distribution, and electrochemical performance.

**Figure 12:**
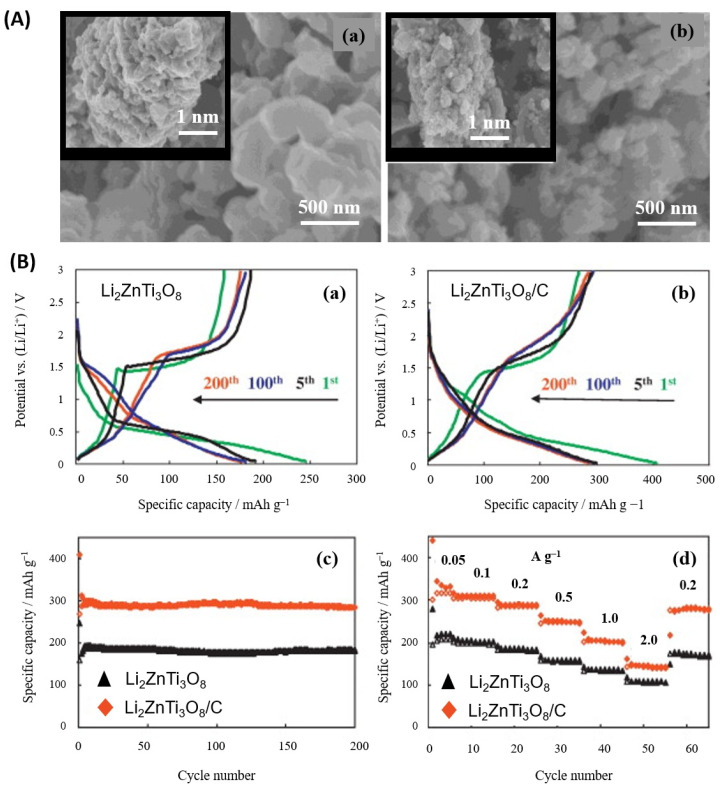
Morphological and electrochemical characterization of Li_2_ZnTi_3_O_8_ and Li_2_ZnTi_3_O_8_/C anodes. (**A**) SEM images of (**a**) Li_2_ZnTi_3_O_8_ and (**b**) Li_2_ZnTi_3_O_8_/C. (**B**) Electrochemical performance, including charge–discharge profiles of (**a**) Li_2_ZnTi_3_O_8_ and (**b**) Li_2_ZnTi_3_O_8_/C, (**c**) cycling performance, and (**d**) rate capability. The Li_2_ZnTi_3_O_8_/C anode exhibits improved cycling stability and rate performance due to carbon coating and reduced Li^+^ diffusion length. Reproduced with permission [147], Elsevier.

In addition to conversion-type metal-oxide anodes, Ti-based insertion anodes have been developed as safer alternatives to graphite because of their stable operating voltage and superior cycling stability. In this context, Figure 12 highlights the role of one-step sol–gel synthesis in improving the electrochemical behavior of Li_2_ZnTi_3_O_8_ through carbon coating. As shown in Figure 12(Aa,Ab), bare Li_2_ZnTi_3_O_8_ consists of relatively larger irregular particles, whereas the Li_2_ZnTi_3_O_8_/C composite exhibits smaller and more homogeneous nanoparticles due to the suppression of particle growth by the carbon layer. HRTEM analysis in the original study confirmed that the Li_2_ZnTi_3_O_8_/C nanoparticles were approximately 20–30 nm in size and coated with a thin carbon layer of about 2–3 nm. This nanosized carbon-coated structure improves electronic conductivity and reduces Li^+^ diffusion distance. Accordingly, Figure 12(Ba,Bb) shows that Li_2_ZnTi_3_O_8_/C maintains a more stable charge–discharge profile than bare Li_2_ZnTi_3_O_8_ during repeated cycling. The cycling and rate data in Figure 12(Bc,Bd) further demonstrate the advantage of carbon coating: Li_2_ZnTi_3_O_8_/C anode retained 284.2 mAh g^−1^ after 200 cycles, compared with 182.9 mAh g^−1^ for bare Li_2_ZnTi_3_O_8_, and delivered better rate capability at high current density. Therefore, this work demonstrates that sol–gel-derived carbon-coated Li_2_ZnTi_3_O_8_ can enhance lithium–ion storage by combining nanoscale particle engineering with improved charge-transfer pathways [147]. Similar structural design principles have been applied to ZnO-based conversion/alloying anodes, where volume expansion and particle pulverization are more severe. The sol–gel-derived ZnO and TiO_2_ core–shell architecture improved cycling stability and Coulombic efficiency by combining the high capacity of ZnO with the structural buffering and kinetic advantages of TiO_2_, delivering 402 mAh g^−1^ over 200 cycles with Coulombic efficiency above 98% [148]. In addition, ZnO/MWCNT free-standing electrodes prepared by sol–gel spin coating further show that infiltrating oxide precursors into a conductive carbon network can preserve electrical contact and suppress mechanical degradation during repeated cycles, with the glycerin-assisted combustion-synthesized ZnO/MWCNT electrode retaining 460 mAh g^−1^ after 100 cycles [149]. These studies collectively indicate that the main advantage of sol–gel synthesis for transition-metal oxide anodes lies not only in low-temperature oxide formation, but also in its ability to engineer multiscale architectures that couple high capacity with improved conductivity, diffusion kinetics, and structural durability.

**Figure 13:**
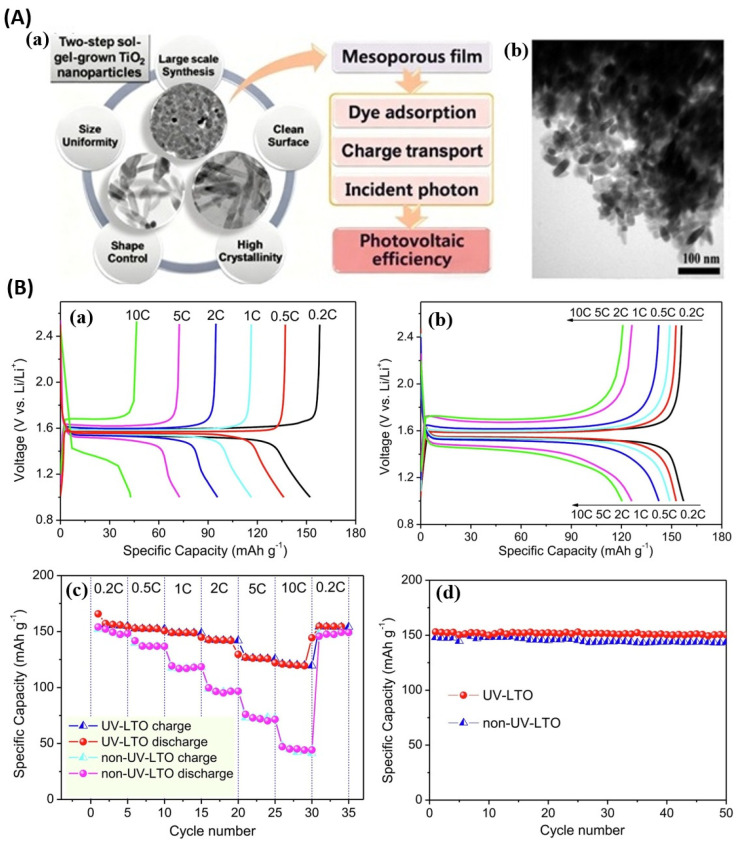
(**A**) Synthesis and morphology of TiO_2_/LTO-based materials: (**a**) Schematic illustration of TiO_2_ nanoparticles prepared by a two-step sol–gel method, showing uniform morphology and size control for photo-energy conversion devices; reproduced with permission [152], Copyright © 2010 American Chemical Society. (**b**) TEM image of TiO_2_ nanoparticles; reproduced with permission [153] Copyright © 2006 American Chemical Society. (**B**) Electrochemical behavior: Initial charge and discharge voltage profiles at different rates for (**a**) non-UV-LTO and (**b**) UV-LTO, (**c**) rate capability, and (**d**) cycling performance comparison of non-UV-LTO and UV-LTO electrodes. Adapted with permission [154], Elsevier.

Ti-based transition-metal oxides, such as TiO_2_ and Li_4_Ti_5_O_12_, represent a distinct class of insertion-type anodes. Their theoretical capacities are much lower than those of conversion-type oxides, but they offer superior structural stability, safety, and long cycle life. Li_4_Ti_5_O_12_ is particularly known as a zero-strain anode because of its negligible volume change during Li^+^ insertion/extraction and its relatively high operating voltage around 1.55 V vs. Li^+^/Li, which reduces severe electrolyte decomposition compared with graphite. However, its low electronic conductivity and slow Li^+^ diffusion require nanosizing, carbon coating, or doping strategies. Modified citric acid sol–gel methods using EDTA-citric acid dual chelation have produced nanoscale Li_4_Ti_5_O_12_ with high phase purity, good dispersion, and long cycle stability [150]. Carbon incorporation is another important advantage of polymeric and chelate–gel precursor routes. Organic chelating agents can serve as complex agents, fuels, dispersants, and carbon sources simultaneously. Under inert or reducing atmospheres, incomplete decomposition of citric acid, glucose, sucrose, or polymeric additives can generate carbon-coated oxide particles. Such in situ carbon coating improves electronic conductivity, suppresses particle aggregation, and buffers mechanical stress during cycling. Kuo and Lin reported a one-pot sol–gel synthesis of Li_4_Ti_5_O_12_/C using citric acid as a carbon source, in which the carbon layer and partial Ti^4+^/Ti^3+^ reduction improved rate capability and cycling stability [151]. To highlight the morphological variations produced by different synthesis approaches, representative TiO_2_ nanostructures are presented in Figure 13(Aa). Among these methods, the sol–gel process is one of the most adaptable and commonly used techniques for fabricating TiO_2_ powders and thin films, offering effective control over phase composition and particle size. In the two-step sol–gel route, uniform anatase TiO_2_ nanoparticles with narrow size distribution, high crystallinity, and clean surfaces were obtained, enabling mesoporous films with improved surface accessibility and charge transport, as shown schematically in Figure 13(Aa) [152]. Titanate nanotube precursors further demonstrated control of morphology through a pH-dependent tube-to-rod transformation, producing single-crystalline anatase TiO_2_ nanorods, as shown in Figure 13(Ab) [153]. These morphology-control concepts are directly relevant to Ti-based insertion anodes such as Li_4_Ti_5_O_12_, where a photo-assisted sol–gel route promoted organic decomposition, formed a more uniform metal–oxygen network, refined particle size, and improved electrochemical kinetics. As shown in Figure 13(Ba–Bd), UV-treated Li_4_Ti_5_O_12_ exhibited better voltage profiles, rate capability, and cycling stability than non-UV-treated Li_4_Ti_5_O_12_, retaining about 120 mAh g^−1^ at 10C and 98.3% capacity after 50 cycles [154].

Overall, sol–gel synthesis of TMO anodes should be understood not merely as a low-temperature route to oxide powders, but as a precursor-engineering strategy. The electrochemical properties of the final anode are strongly affected by the chelating agent, metal-to-ligand ratio, pH, solvent, gelation temperature, drying conditions, calcination atmosphere, and heat-treatment temperature. A summary of the electrochemical performances and morphologies of various transition metal oxide anode materials synthesized by sol–gel or modified sol–gel methods is presented in Table 2. For conversion-type oxides, the main design goal is to generate nanosized, porous, and electrically connected architectures that can accommodate conversion-induced volume changes. For mixed spinel oxides, homogeneous cation distribution and phase purity are critical. For insertion-type Ti-based oxides, sol–gel routes are useful for nanosizing, carbon coating, and dopant incorporation. Therefore, polymeric and chelate-gel precursor chemistry provides a versatile platform for designing TMO anodes with improved capacity, rate capability, and cycling stability. However, several challenges remain. First, the high surface area of sol–gel-assisted nanoparticles often leads to increased electrolyte decomposition and irreversible capacity loss during the first cycle. Second, excessive carbon residues or incomplete decomposition of organic precursors can reduce tap density and complicate phase identification. Third, although laboratory-scale sol–gel synthesis provides excellent compositional control, scale-up requires careful management of gel homogeneity, combustion exothermicity, gas evolution, and batch-to-batch reproducibility. Future work should therefore focus on balancing nanoscale reactivity with electrode-level requirements, including high mass loading, high tap density, low irreversible capacity, and stable interfacial chemistry.

## 4. Conclusions, Challenges, and Future Direction

In summary, gel-based synthesis techniques—such as traditional sol–gel, Pechini, and chelate–nitrate methods—provide several key advantages for developing high-performance anode materials for lithium–ion batteries. A major benefit is the ability to achieve homogeneous mixing of metal precursors at the molecular level within the gel matrix, ensuring precise stoichiometric control and the production of phase-pure, compositionally accurate materials. This level of homogeneity is challenging to achieve with conventional solid-state or co-precipitation methods. Additionally, gel-based approaches typically require lower synthesis temperatures (~300–600 °C) compared to traditional ceramic methods, which reduces energy consumption, minimizes grain coarsening, and preserves nanoscale features. Another significant advantage is the ability to tailor morphology through the gel network by modifying chelating agents, pH, polymer-to-metal ratio, or drying techniques (e.g., supercritical or freeze-drying), enabling control over pore structure, particle size, and surface area—parameters that impact lithium–ion diffusion and electrolyte accessibility. Furthermore, gel systems are well-suited for creating composites, as conductive materials like carbon black, carbon nanotubes (CNTs), or graphene can be incorporated directly into the sol or gel phase before calcination, ensuring uniform distribution of conductive networks within the active material to improve electronic conductivity and structural stability during cycling. In summary, the versatility, molecular-level control, and capacity to produce nanostructured, porous, and conductive composites make gel-based synthesis an effective approach for developing advanced anode materials.

Despite these advantages, gel-based synthesis routes are not without their limitations. A significant challenge is the potential formation of phase impurities or carbonaceous residues caused by incomplete combustion of organic components or uneven gel drying. Poor gelation conditions, excessive citric acid, or insufficient oxidizing strength of nitrate salts can result in unreacted organics or the formation of mixed-valence oxide phases, which can negatively impact electrochemical performance. In combustion-based processes, managing the exothermic reaction during calcination is crucial; excessively high temperatures can cause particle sintering, grain growth, and porosity loss, undermining the advantages of nanoscale structures. Furthermore, ensuring batch-to-batch reproducibility can be difficult, particularly when scaling up from laboratory to industrial production, as gel viscosity, solvent evaporation rates, and gelation time are highly sensitive to environmental and compositional factors. Environmental and safety concerns also pose challenges. Many gel routes involve the use of toxic or volatile precursors, such as nitrate salts, ethylene glycol, or ethanol, which can generate harmful by-products (e.g., NOₓ gases) during thermal decomposition. This necessitates careful handling and appropriate ventilation or scrubbing systems, particularly for industrial-scale production. Furthermore, maintaining gel stability over extended periods is often difficult; premature aging, phase separation, or syneresis (gel shrinkage) can occur, affecting the consistency of the final product. Therefore, while gel-based methods offer unique advantages in terms of morphology control, homogeneity, and energy efficiency, their optimization for scalability, environmental compatibility, and product consistency remains a key area for further research and process engineering.

Future research on gel-based synthesis of lithium–ion battery anode materials is expected to focus on improving scalability, environmental sustainability, and precise structural engineering. For TMOs such as Fe_3_O_4_, MnO_x_, TiO_2_, NiO, and multicomponent oxides, next-generation polymeric and chelate gel routes should emphasize heteroatom doped conductive network integration to produce hierarchically porous nanostructures with optimized phase purity and interfacial stability. The incorporation of in situ carbonization, graphene, carbon nanotubes, and heteroatom-doped carbon matrices will be increasingly important to enhance electronic conductivity and buffer volume changes associated with conversion reactions. In silicon and SiO_x_ anodes, future gel strategies are likely to concentrate on the fabrication of yolk–shell, aerogel, and multiscale porous Si/C or SiO_x_/C composites. Advanced precursor chemistry using a combination of silicon alkoxides (TEOS, TMOS), biopolymer-derived carbon sources, and nitrogen- or phosphorus-containing ligands will enable simultaneous control of porosity, heteroatom doping, and artificial interphase formation. Moreover, environmentally benign aqueous sol–gel systems, the adoption of greener precursors, low-toxicity chelating agents, and NOₓ-minimized combustion protocols will be essential for sustainable manufacturing. The integration of rheological phase control and freeze-drying, combined with industrial techniques such as spray-drying and 3D printing, may further enable continuous and industrially scalable production of structurally uniform electrode materials.

## Figures and Tables

**Table 1:** The electrochemical performance and morphology of various Si-based anodes in Li–ion batteries synthesized using sol–gel or modified sol–gel methods.

Materials /Methods	Chelating Agent/ Precursor	Morphology	Electrochemical Performance		Ref.
			Specific Capacity/C Density	Capacity Retention	
bare Si Si/TiO_2_ (Sol–gel)	Commercial Si powder	Microsized Si (0.4–3 μm)	~2000 mAh g^–1^ @3.5 A g^–1^	4.5% (900th cycles)	[79]
	Si powder (0.4–3 μm), Ti(OCH(CH_3_)_2_)_4_	Microsized Si covered by TiO_2_ nanoparticle	~1625 mAh g^–1^ @3.5 A g^–1^	43.6% (900th cycles)	
bare Si Si/SiO_x_ (Sol–gel)	Commercial Si nanopowder	Si nanoparticles	~2850 mAh g^–1^ @0.2 A g^–1^	16.8% (100th cycles)	[80]
	Si nano (50–100 nm) powder, (C_2_H_5_O)_3_SiH	Si nanocrystal-embedded Si/SiO_x_ nanocomposite	~1914 mAh g^–1^ @0.2 A g^–1^	78.3% (100th cycles)	
bare Si Si/C hybrid (Sol–gel)	Commercial Si powder	agglomerated Microsized	~2617 mAh g^–1^ @0.2 A g^–1^	12.1% (at 200th cycles)	[81]
	TEOS ⟶ silica aerogel, Mg powder, Chlorotrimethylsilane	3D interconnected Carbon-coated porous Si	~2269 mAh g^–1^ @0.2 A g^–1^	68.4% (200th cycles)	
bare Si C/Si C@TiO_2_@Si (CTS) (Sol–gel)	Si nanopowder	Si nanoparticles	~2492.9 mAh g^–1^ @0.1 A g^–1^	0.24% (50th cycles)	[82]
	Si, TEOS, Dopamine hydrochloride, Ethylene glycol	Carbon coated Si sphere	~680.2 mAh g^–1^ @0.1 A g^–1^	28.6% (50th cycles	
	Si, TEOS, Tetra butyl titanate, Dopamine hydrochloride, Ethylene glycol	Yolk–shell structure of carbon@TiO_2_@Si	~980.9 mAh g^–1^ @0.1 A g^–1^	39.8% (50th cycles)	
SiO_x_@C (Pechini)	Tetramethyl orthosilicate, 3-aminophenol, formaldehyde, CTAB	Core–shell structured spherical particle	~509.2 mAh g^–1^ @0.1 A g^–1^	80% (100th cycles)	[83]
Crystalline Si (Chelate gel)	Si-waste (Coal-fly ash), Citric acid/lactic acide	Micron-sized Si secondary particle consists of submicron-sized primary particles	~1250 mAh g^–1^ @0.2 A g^–1^	24.92% (50th cycles)	[86]
Si@BaTiOx (Pechini)	Si nanopowder, ethylene glycol, BaOH·H_2_O, Ti(OC_4_H_9_)_4_	Amorphous BaTiOx and carbon coated Si nanoparticles	~2000 mAh g^–1^ @1 C	65% (600th cycles) (at 60 °C)	[88]
Si/SiO_x_/C/Graphene (Sol–gel)	TEOS, CTAB, Resorcinol-formaldehyde resin, graphene suspension	Carbon-coated Si/SiO_x_ nanosphere anchored on graphene sheets	~773.2 mAh g^–1^ @0.3 A g^–1^	79.5% (200th cycles)	[90]
Layered graphene/SiOC hetrostructure	N-doped Graphene oxide aerogel, methylphenylvinylhydrogen polysiloxane	SiOC embedded in Porous sandwiched multilayer graphene sheet	~751 mAh g^–1^ @37 mA g^–1^	95% (1000th cycles)	[91]
Si/SiO_x_ nanosphere (Sol–gel)	Triethoxysilane, Coal-tar pitch (carbon source)	Agglomerated Nanosphere (100–200 nm)	~951.4 mAh g^–1^ @0.2 A g^–1^	78% (100th cycles)	[92]
2D Si/SiO_x_ nanofoils (sol–gel)	Trichlorosilane	Nanofoils	~ 739.8 mAh g^–1^ @1 A g^–1^	90.6% (200th cycles)	[93]
Si/SiO nanocomposite (Sol–gel)	Tetraethoxysilane (TEOS)	Spherical particle with average size of about 50 nm	~ 827.3 mAh g^–1^ @1 A g^–1^	65% (20th cycles)	[96]
Si@SiO_2_@C (Sol–gel)	TEOS, Polydopamine	Yolk–shell structure	~ 1500 mAh g^–1^ @4.2 A g^–1^	74% (1000th cycles)	[97]
SiO_x_/C microspheres (Sol–gel)	Vinyltriethoxysilane, 3-mercaptopropyltrimethoxysilane,3-thiocyanatopropyltriethoxysilane	Yolk–shell structured SiO_x_/C microspheres	~ 1165 mAh g^–1^ @0.1 A g^–1^	84% (150th cycles)	[98]
Si nanoparticle Carbon coated Si@10C (Sol–gel coating)	Commercial Si particle	Nanoparticle (~80 nm)	~2254 mAh g^–1^ @0.5 A g^–1^	20% (40th cycles)	[99]
	Resorcinol-for- maldehyde resin (RF) polymer, CTAB	C-coated nanoparticles (~80–200 nm)	~3394 mAh g^–1^ @0.5 A g^–1^	66% (200th cycles)	
Si@C@TiO_2_ (Sol–gel coating)	RF resin polymer, Si nanoparticle, CTAB surfactant	Core−shell−shell nanoparticles	~1174.4 mAh g^–1^ @0.42 A g^–1^	86% (710th cycles)	[105]
Mesoporous Silicon (Sol–gel)	Tetraethyl orthosilicate, Poly(butadiene-b-ethylene oxide)	Nanostructure with roughly spherical pores	~1750 mAh g^–1^ @5 A g^–1^	73.8% (500th cycles)	[107]
CNT/meso-SiO_2_ nanocomposite (Sol–gel)	CNT sponges, TEOS, CTAB template	Hierarchical porous structure	~2100 mAh g^–1^ @0.5 A g^–1^	85.7% (500th cycles)	[108]
Si@TiO_2_@Graphene (Sol–gel)	Porous Si, Resorcinol-formaldehyde resin polymer, CTAB	Graphene nanoribbons (GNRs) enlacing Si@TiO_2_ nanoparticles	~1684.6 mAh g^–1^ @0.5 A g^–1^	76.8% (300th cycles)	[111]
Si@B-C (B doping via Sol–gel)	Si nanoparticles, boric acid, CTAB, RF polymer	Peanut-like core–shell architecture	~906.7 mAh g^–1^ @0.5 A g^–1^	89% (500th cycles)	[112]
Si@C@Ge (Ge doping via Sol–gel coating)	Si nanoparticles, TEOG, CTAB, RF ((resorcinol–formaldehyde) polymer	Inner silicon core (≈60–100 nm), carbon layer (20–30 nm), Ge nanoparticles on the surface	~2085 mAh g^–1^ @0.5 A g^–1^	83.3% (150th cycles)	[113]
TiO_2_-coated SiO (Sol–gel coating)	(Ti(OCH(CH_3_)_2_)_4_, SiO powder,	50 to 150 nm TiO_2_ coating layer on micro SiO	~1000 mAh g^–1^ @0.1 A g^–1^	-------	[124]
Si@a-TiO_2_ (Sol–gel coating)	Titanium isopropylate, Si nanoparticles	Si nanoparticles coated by TiO_2_ shell	~ 3061 mAh g^–1^ @0.42 A g^–1^	56.1% (200th cycles)	[128]

**Table 2:** The electrochemical performance and morphology of various transition metal oxide anodes in Li–ion batteries synthesized using sol–gel or modified sol–gel methods.

Materials /Methods	Chelating Agent/ Precursor	Morphology (Particle Size)	Electrochemical Performance		Ref.
			Specific Capacity/C Density	Capacity Retention	
Co_3_O_4_ octahedra (Sol–gel)	Pluromic type triblock copolymer, PEO–PPO–PEO (F127)	Mesoporous octahedra (1–2 μm)	~1567 mAh g^–1^ @0.2 A g^–1^	~76.5% (60th cycles)	[141]
Fe_2_O_3_-CF composites (Sol–gel)	Fe(NO_3_)_3_·9H_2_O, N,N-dimethylformamide (DMF	Needle-like Fe_2_O_3_ core (10 nm to 50 nm)/carbon fiber sheath architecture	~946 mAh g^–1^ @0.05 A g^–1^	~67.0% (150th cycles)	[142]
Fe_3_O_4_/C nanocomposite (Sol–gel)	Fe_2_O_3_ xerogel precursor from FeCl_3_, propylene oxide	Fe_3_O_4_ nanoparticles coated with carbon layer.	~850 mAh g^–1^ @0.1 A g^–1^	~97.0% (60th cycles)	[143]
F doped-Fe_3_O_4_ (Pechini)	Ferric nitrate hydrate, Iron (III) fluoride, Ethylene glycol (EG)	Spherical morphology with particles size ~30 nm	~902.3 mAh g^–1^ @0.2 A g^–1^	~81.7% (100th cycles)	[144]
ZnFe_2_O_4_ (Combustion)	Zinc acetate, iron acetate and citric acid	Nanoparticles (~100–200 nm)	~1312 mAh g^–1^ @0.1 A g^–1^	~95% (50th cycles)	[145]
ZnMn_2_O_4_ (Combustion)	Zinc nitrate, manganese nitrate and citric acid	block-like sub-micron particles (0.48–1.5 μm)	~1100.2 mAh g^–1^ @0.1 A g^–1^	~25% (40th cycles)	[146]
Li_2_ZnTi_3_O_8_/C (Sol–gel)	Ti{OCH(CH_3_)_2_}_4_, zinc acetate, Li_2_CO_3_	Nanoparticles (20–30 nm)	~408 mAh g^–1^ @0.2 A g^–1^	~69.6% (200th cycles)	[147]
ZnO/TiO_2_ (Sol–gel)	Ti{OCH(CH_3_)_2_}_4_, ZnO nanopowder	Core–shell nanocomposites	~750 mAh g^–1^ @0.45 A g^–1^	~81.3% (1000th cycles)	[148]
ZnO/MWCNT (Sol–gel)	Triethanolamine (TEA), Glycerin (GLY), Zinc acetate	ZnO/MWCNT core–shell units (50–70 nm)	~852 mAh g^–1^ @0.45 A g^–1^	~54% (100th cycles)	[149]
Li_4_Ti_5_O_12_ (Citrate–gel)	Ethylene diamine tetraacetic acid (EDTA) and citric acid, Ti(C_4_H_9_O)_4_	Nanoparticles (~50–100 nm)	~164 mAh g^–1^ @1 C	~97% (1000th cycles)	[150]
Li_4_Ti_5_O_12_/C (Citrate–gel)	CH_3_COOLi·2H_2_O, citric acid, Ti(C_4_H_9_O)_4_	Nanoparticles (~100–300 nm)	~147.9 mAh g^–1^ @20 C	~98% (50th cycles)	[151]
Li_4_Ti_5_O_12_ (Photo-assisted sol–gel)	Acetylacetone C_16_H_36_O_4_Ti, CH_3_COOLi· 2H_2_O	Spherical nanoparticles (~87.7 nm)	~152.6 mAh g^–1^ @0.2 C	~98.3% (50th cycles)	[154]
NiFe_2_O_4_ (citrate–gel)	Citric acid, Nickel nitrate, Iron nitrate	Spherical nanoparticles (~20–40 nm)	~923 mAh g^–1^ @0.5 A g^–1^	~85% (100th cycles)	[155]
Zn_0.5_Mg_0.5_ FeMnO_4_ (citrate–gel)	Citric acid, ZnCl_2_, metal (Mg, Fe, Mn) nitrate salts	Microsized porous oxide (pore sizes ≈ 2 to 5 μm)	~484.1 mAh g^–1^ @0.15 A g^–1^	~62% (300th cycles)	[156]
NiCo_2_O_4_ (Pechini)	Citric acid, Nickel nitrate, Cobalt nitrate, EG	Spherical nanoparticles (~5–10 nm)	~860 mAh g^–1^ @0.5 A g^–1^	~95% (200th cycles)	[56]
MnO/C (Combustion)	Manganese nitrate-cellulose combustion	MnO (~5 nm) Nanoparticles embedded in porous carbon	~860 mAh g^–1^ @0.2 A g^–1^	~95% (90th cycles)	[157]
MnO/N–C (Combustion)	Cotton-template glycine–nitrate salt combustion	MnO (~5 nm) Nanoparticles dispersed in amorphous carbon	~710 mAh g^–1^ @0.5 A g^–1^	~81.6% (100th cycles)	[158]

## Data Availability

No new data were created in this study.

## References

[1] Dunn B., Kamath H., Tarascon J. (2011). For the Grid: A Battery of Choices. Science.

[2] Parvizi P., Jalilian M., Amidi A.M., Zangeneh M.R., Riba J. (2025). From Present Innovations to Future Potential: The Promising Journey of Lithium-Ion Batteries. Micromachines.

[3] Moshtev R., Johnson B. (2000). State of the Art of Commercial Li Ion Batteries. J. Power Sources.

[4] Mohamed N., Allam N.K. (2020). Recent Advances in the Design of Cathode Materials for Li-Ion Batteries. RSC Adv..

[5] Chang H., Wu Y., Han X., Yi T. (2021). Recent Developments in Advanced Anode Materials for Lithium-Ion Batteries. Energy Mater..

[6] Islam M., Ur S., Yoon M. (2015). Improved Performance of Porous LiFePO_4_/C as Lithium Battery Cathode Processed by High Energy Milling Comparison with Conventional Ball Milling. Curr. Appl. Phys..

[7] Ali B., Muhammad R., Moeez I., Park J., Islam M., Cho M. (2024). Improving High-Rate and Long-Life Cycling of Li_4_Ti_5_O_12_ Anode by Dual Doping of Cd^2+^ and Ge^4+^. Adv. Sustain. Syst..

[8] Cui Y. (2021). Silicon Anodes. Nat. Energy.

[9] Seok M., Heo I., Kim S., Yang J., Kim J., Min S., Chae J., Cheol W. (2022). High-Areal-Capacity of Micron-Sized Silicon Anodes in Lithium-Ion Batteries by Using Wrinkled-Multilayered-Graphenes. Energy Storage Mater..

[10] Wu H., Cui Y. (2012). Designing Nanostructured Si Anodes for High Energy. Nano Today.

[11] Cabana J., Monconduit L., Larcher D., Palacín M.R. (2010). Beyond Intercalation-Based Li-Ion Batteries: The State of the Art and Challenges of Electrode Materials Reacting through Conversion Reactions. Adv. Mater..

[12] Xu K. (2014). Electrolytes and Interphases in Li-Ion Batteries and Beyond. Chem. Rev..

[13] Reddy M.V., Subba Rao G.V., Chowdari B.V.R. (2013). Metal Oxides and Oxysalts as Anode Materials for Li Ion Batteries. Chem. Rev..

[14] Mishra A.K., Monika, Anjali, Patial B.S. (2025). Si as an Anode Material in Li-Ion Batteries—A Review. Future Batter..

[15] Zhang Z., Wu Y., Mo Z., Lei X., Xie X., Xue X., Qin H., Jiang H. (2025). Research Progress of Silicon-Based Anode Materials for Lithium-Ion Batteries. RSC Adv..

[16] Luo W., Chen X., Xia Y., Chen M., Wang L., Wang Q., Li W., Yang J. (2017). Surface and Interface Engineering of Silicon-Based Anode Materials for Lithium-Ion Batteries. Adv. Energy Mater..

[17] Park I., Lee H., Chae O.B. (2024). Synthesis Methods of Si/C Composite Materials for Lithium-Ion Batteries. Batteries.

[18] Khan M., Yan S., Ali M., Mahmood F., Zheng Y., Li G., Liu J., Song X., Wang Y. (2024). Innovative Solutions for High-Performance Silicon Anodes in Lithium-Ion Batteries: Overcoming Challenges and Real-World Applications. Nano-Micro Lett..

[19] Lu Y., Yu L., Lou X.W. (2018). (David) Nanostructured Conversion-Type Anode Materials for Advanced Lithium-Ion Batteries. Chem.

[20] Cao K., Jin T., Yang L., Jiao L. (2017). Recent Progress in Conversion Reaction Metal Oxide Anodes for Li-Ion Batteries. Mater. Chem. Front..

[21] Yu S.H., Lee S.H., Lee D.J., Sung Y.E., Hyeon T. (2016). Conversion Reaction-Based Oxide Nanomaterials for Lithium Ion Battery Anodes. Small.

[22] Zhao C., Yao S., Li C., An Y., Zhao S., Sun X., Wang K., Zhang X., Ma Y. (2024). Recent Advances in Transition Metal Oxides as Anode Materials for High-Performance Lithium-Ion Capacitors. Chem. Eng. J..

[23] Mersian H., Alizadeh M. (2020). Effect of Diverse Pechini Sol-Gel Parameters on the Size, Morphology, Structural and Optical Properties of the Tenorite (CuO) NPs: A Facile Approach for Desired Properties. Ceram. Int..

[24] Navas D., Fuentes S., Castro-Alvarez A., Chavez-Angel E. (2021). Review on Sol-Gel Synthesis of Perovskite and Oxide Nanomaterials. Gels.

[25] Kakihana M. (1996). “Sol-Gel” Preparation of High Temperature Superconducting Oxides. J. Sol-Gel Sci. Technol..

[26] Majeed A.H., Mohammed I.K., Mohammed L.A., Hammoodi O.G., Alheety M.A. (2026). Recent Advances in Chelating Polymers: Synthesis, Properties, and Applications. React. Funct. Polym..

[27] Patil K.C., Aruna S.T., Mimani T. (2002). Combustion Synthesis: An Update. Curr. Opin. Solid State Mater. Sci..

[28] Sang J., Sun C., Zheng H., Wang Q. (2025). Algae-Derived Gel Carbon Framework Encapsulating Si@NiO/Ni Quantum Dots to Construct High-Speed Electron Transport Channels for Lithium-Ion Battery Anodes. J. Energy Storage.

[29] Han W.K., Ko Y.N., Yoon C.S., Choa Y.H., Oh S.T., Kang S.G. (2009). Synthesis and Characterization of Hollow Silicon-Carbon Composites as a Lithium Battery Anode Material. Korean J. Mater. Res..

[30] Saidi N.M., Abdah M.A.A.M., Mustafa M.N., Walvekar R., Khalid M., Khosla A. (2025). Advancements in Silicon Anodes for Enhanced Lithium-Ion Batteries Performance: Innovations Toward Next-Gen Superbatteries. Batter. Energy.

[31] Su P., Zhou Y., Wu J., Shao J., Shen L., Bao N. (2024). Flexible Silicon/Titanium Dioxide/Reduced Graphene Oxide Self-Standing Electrode with High Performance and High Stability for Lithium-Ion Batteries. Ind. Eng. Chem. Res..

[32] Sun R., Mai Y., Luo M., Cheng Y., Feng L., Dai Y., Wu F. Calcium Ion Induced Conductive Hydrogel Coating on Silicon Anode Material for Enhanced Lithium-Ion Batteries Performance. https://papers.ssrn.com/sol3/papers.cfm?abstract_id=5061245.

[33] Brinker C.J., Scherer G.W. (1990). Sol-Gel Science: The Physics and Chemistry of Sol-Gel Processing.

[34] Livage J., Beteille F., Roux C., Chatry M., Davidson P. (1998). Sol-Gel Synthesis of Oxide Materials. Acta Mater..

[35] Hench L.L., West J.O.N.K. (1990). The Sol-Gel Process. Chem. Rev..

[36] Pierre A.C., Pajonk G.M. (2002). Chemistry of Aerogels and Their Applications. Chem. Rev..

[37] Cushing B.L., Kolesnichenko V.L., O’Connor C.J. (2004). Recent Advances in the Liquid-Phase Syntheses of Inorganic Nanoparticles. Chem. Rev..

[38] Aricò A.S., Bruce P., Scrosati B., Tarascon J.M., Van Schalkwijk W. (2005). Nanostructured Materials for Advanced Energy Conversion and Storage Devices. Nat. Mater..

[39] Hanaor D.A.H., Sorrell C.C. (2011). Review of the Anatase to Rutile Phase Transformation. J. Mater. Sci..

[40] Mahshid S., Askari M., Ghamsari M.S. (2007). Synthesis of TiO_2_ Nanoparticles by Hydrolysis and Peptization of Titanium Isopropoxide Solution. J. Mater. Process. Technol..

[41] Armstrong A.R., Armstrong G., Canales J., Bruce P.G. (2005). TiO_2_-B Nanowires as Negative Electrodes for Rechargeable Lithium Batteries. J. Power Sources.

[42] Fujishima A., Zhang X., Tryk D.A. (2008). TiO_2_ Photocatalysis and Related Surface Phenomena. Surf. Sci. Rep..

[43] Sun S., Zeng H. (2002). Size-Controlled Synthesis of Magnetite Nanoparticles. J. Am. Chem. Soc..

[44] Gupta A.K., Gupta M. (2005). Synthesis and Surface Engineering of Iron Oxide Nanoparticles for Biomedical Applications. Biomaterials.

[45] Ansari A.A., Ahmad N., Alam M., Adil S.F., Ramay S.M., Albadri A., Ahmad A., Al-Enizi A.M., Alrayes B.F., Assal M.E. (2019). Physico-Chemical Properties and Catalytic Activity of the Sol-Gel Prepared Ce-Ion Doped LaMnO_3_ Perovskites. Sci. Rep..

[46] Yang J., Xu J.J. (2004). Nonaqueous Sol-Gel Synthesis of High-Performance LiFePO_4_. Electrochem. Solid-State Lett..

[47] Livage J., Henry M., Sanchez C. (1988). Sol-Gel Chemistry of Transition Metal Oxides. Prog. Solid State Chem..

[48] Sanchez C., Julián B., Belleville P., Popall M. (2005). Applications of Hybrid Organic-Inorganic Nanocomposites. J. Mater. Chem..

[49] Yarbrough R., Davis K., Dawood S., Rathnayake H. (2020). A Sol-Gel Synthesis to Prepare Size and Shape-Controlled Mesoporous Nanostructures of Binary (II-VI) Metal Oxides. RSC Adv..

[50] Dimesso L., Klein L., Aparicio M., Jitianu A. (2018). Pechini Processes: An Alternate Approach of the Sol-Gel Method, Preparation, Properties, and Applications. Handbook of Sol-Gel Science and Technology.

[51] Pechini M.P. (1967). Method of Preparing Lead and Alkaline Earth Titanates and Niobates and Coating Method Using the Same to Form a Capacitor. U.S. Patent.

[52] Myasoedova T.N., Kalusulingam R., Mikhailova T.S. (2022). Sol-Gel Materials for Electrochemical Applications: Recent Advances. Coatings.

[53] Yao Z., Sasaki U., Zhang X., Liu L. (2025). Pechini Method Revisited: From Synthesis Innovation to Multifunctional Materials. Chem. Mater..

[54] Mosa J., Aparicio M. (2020). Sol-Gel Synthesis of Nanocrystalline Mesoporous Li_4_Ti_5_O_12_ Thin-Films as Anodes for Li-Ion Microbatteries. Nanomaterials.

[55] Moradi H., Eshaghi A., Hosseini S.R., Ghani K. (2016). Fabrication of Fe-Doped TiO_2_ Nanoparticles and Investigation of Photocatalytic Decolorization of Reactive Red 198 under Visible Light Irradiation. Ultrason. Sonochem..

[56] Islam M., Ali G., Jeong M.G., Chung K.Y., Nam K.W., Jung H.G. (2021). Electrochemical Storage Behavior of NiCo_2_O_4_ Nanoparticles Anode with Structural and Morphological Evolution in Lithium-Ion and Sodium-Ion Batteries. Int. J. Energy Res..

[57] Wang Z., Ai L., Ding J., Zhu P., Zhuang J., Zhao J., Li B., Yu F., Duan X. (2022). MgMn_2_O_4_ Anode Materials for Lithium-Ion Batteries: Synthesis and Cationic Distribution Study. Vacuum.

[58] Julien C.M., Mauger A. (2024). Fabrication of Li_4_Ti_5_O_12_ (LTO) as Anode Material for Li-Ion Batteries. Micromachines.

[59] Zhao T., Zhang X., Liu Z., Gu Q., Jin X., Xie S., Liu S. (2024). N-Doped Porous Carbon Encapsulated MnFe_2_O_4_ Nanoparticles as Advanced Anodes for Li-Ion Batteries. Electron. Mater. Lett..

[60] Hu C., Guo J., Wen J., Peng Y. (2013). Preparation and Electrochemical Performance of Nano-Co_3_O_4_ Anode Materials from Spent Li-Ion Batteries for Lithium-Ion Batteries. J. Mater. Sci. Technol..

[61] Rodríguez F.A., Rivero E.P., González I. (2018). Adapted Pechini Method to Prepare DSA Type Electrodes of RuO_2_-ZrO_2_ Doped with Sb_2_O_5_ over Titanium Plates. MethodsX.

[62] Jain S.R., Adiga K.C., Pai Verneker V.R. (1981). A New Approach to Thermochemical Calculations of Condensed Fuel-Oxidizer Mixtures. Combust. Flame.

[63] Mulik C.U., Kamat R.S., Wang X., Padwal C., Chougale M.Y., Dubal D.P., Jadhav L.D. (2024). High-Performance Li-Ion Battery Cathode: Mn-Doped LiFePO_4_ via Solution Combustion Synthesis Method. J. Electroanal. Chem..

[64] Varma A., Mukasyan A.S., Rogachev A.S., Manukyan K.V. (2016). Solution Combustion Synthesis of Nanoscale Materials. Chem. Rev..

[65] Padayatchee S., Ibrahim H., Friedrich H.B., Olivier E.J., Ntola P. (2025). Solution Combustion Synthesis for Various Applications: A Review of the Mixed-Fuel Approach. Fluids.

[66] De Sloovere D., Marchal W., Ulu F., Vranken T., Verheijen M., Van Bael M.K., Hardy A. (2017). Combustion Synthesis as a Low Temperature Route to Li_4_Ti_5_O_12_ Based Powders for Lithium-Ion Battery Anodes. RSC Adv..

[67] Zhang P., Fan H., Fu Y., Li Z., Deng Y. (2006). Synthesis and Electrochemical Properties of Sol-Gel Derived LiMn_2_O_4_ Cathode for Lithium-Ion Batteries. Rare Met..

[68] Bruce P.G., Scrosati B., Tarascon J.M. (2008). Nanomaterials for Rechargeable Lithium Batteries. Angew. Chemie Int. Ed..

[69] Stella K.C., Nesaraj A.S. (2010). Effect of Fuels on the Combustion Synthesis of NiAL_2_O_4_ Spinel Particles. Iran. J. Mater. Sci. Eng..

[70] Sherikar B.N., Sahoo B., Umarji A.M. (2020). Effect of Fuel and Fuel to Oxidizer Ratio in Solution Combustion Synthesis of Nanoceramic Powders: MgO, CaO and ZnO. Solid State Sci..

[71] Ianoş R., Băbuţă R., Păcurariu C., Lazău R., Istratie R., Butaciu C. (2017). Combustion Synthesis of ZnAl_2_O_4_ Powders with Tuned Surface Area. Ceram. Int..

[72] Huang W., Wang G., Luo C., Xu Y., Xu Y., Eckstein B.J., Chen Y., Wang B., Huang J., Kang Y. (2019). Controllable Growth of LiMn_2_O_4_ by Carbohydrate-Assisted Combustion Synthesis for High Performance Li-Ion Batteries. Nano Energy.

[73] Zhu C., Akiyama T. (2014). Optimized Conditions for Glycine-Nitrate-Based Solution Combustion Synthesis of LiNi_0.5_Mn_1.5_O_4_ as a High-Voltage Cathode Material for Lithium-Ion Batteries. Electrochim. Acta.

[74] Michalska M., Ławniczak P., Strachowski T., Ostrowski A., Bednarski W. (2022). Structural Studies and Selected Physical Investigations of LiCoO_2_ Obtained by Combustion Synthesis. Beilstein J. Nanotechnol..

[75] Li X., Shi H., Zhang L., Chen J., Lü P. (2020). Novel Synthesis of SiO_x_/C Composite as High-Capacity Lithium-Ion Battery Anode from Silica-Carbon Binary Xerogel. Chin. J. Chem. Eng..

[76] Jeong M.G., Islam M., Du H.L., Lee Y.S., Sun H.H., Choi W., Lee J.K., Chung K.Y., Jung H.G. (2016). Nitrogen-Doped Carbon Coated Porous Silicon as High Performance Anode Material for Lithium-Ion Batteries. Electrochim. Acta.

[77] Jeong M.G., Du H.L., Islam M., Lee J.K., Sun Y.K., Jung H.G. (2017). Self-Rearrangement of Silicon Nanoparticles Embedded in Micro-Carbon Sphere Framework for High-Energy and Long-Life Lithium-Ion Batteries. Nano Lett..

[78] Saleem M., Lassi U., Srivastava V., Tuomikoski S. (2025). A Review of Silicon-Carbon Anode Materials: The Role of Precursor and Its Effect on Lithium-Ion Battery Performance. J. Power Sources.

[79] Usui H., Wasada K., Shimizu M., Sakaguchi H. (2013). TiO_2_/Si composites synthesized by sol–gel method and their improved electrode performance as Li-ion battery anodes. Electrochim. Acta.

[80] Park E., Yoo H., Lee J., Park M.-S., Kim Y.-J., Kim H. (2015). Dual-Size Silicon Nanocrystal Embedded SiO_x_ Nanocomposite as a High-Capacity Lithium Storage Material. ACS Nano.

[81] Li Q., Yin L., Gao X. (2015). Reduction Chemical Reaction Synthesized Scalable 3D Porous Silicon/Carbon Hybrid Architectures as Anode Materials for Lithium Ion Batteries with Enhanced Electrochemical Performance. RSC Adv..

[82] Wang K., Li N., Xie J., Lei G., Song C., Wang S., Dai P., Liu X., Zhang J., Guo X. (2021). Dual Confinement of Carbon/TiO_2_ Hollow Shells Enables Improved Lithium Storage of Si Nanoparticles. Electrochim. Acta.

[83] Cao K.L.A., Arif A.F., Kamikubo K., Izawa T., Iwasaki H., Ogi T. (2019). Controllable Synthesis of Carbon-Coated SiO_x_ Particles through a Simultaneous Reaction between the Hydrolysis-Condensation of Tetramethyl Orthosilicate and the Polymerization of 3-Aminophenol. Langmuir.

[84] Kim T.G., An G.S., Han J.S., Hur J.U., Park B.G., Choi S.C. (2017). Synthesis of Size Controlled Spherical Silica Nanoparticles via Sol-Gel Process within Hydrophilic Solvent. J. Korean Ceram. Soc..

[85] Kaur H., Chaudhary S., Kaur H., Chaudhary M., Jena K.C. (2022). Hydrolysis and Condensation of Tetraethyl Orthosilicate at the Air–Aqueous Interface: Implications for Silica Nanoparticle Formation. ACS Appl. Nano Mater..

[86] Yudha C.S., Apriliyani E., Arinawati M., Paramitha T. (2024). Carboxylic Acid Assisted Synthesis of Crystalline Silicon Derived from Coal Fly-Ash for Li-Ion Batteries Anode Material. Evergreen.

[87] Yang H.W., Lee J.H., Jung S.C., Myung S.T., Kang W.S., Kim S.J. (2016). Fabrication of Si/SiO_x_ Anode Materials by a Solution Reaction-Based Method for Lithium Ion Batteries. J. Korean Inst. Met. Mater..

[88] Park H., Choi S., Lee S.J., Cho Y.G., Hwang G., Song H.K., Choi N.S., Park S. (2016). Design of an Ultra-Durable Silicon-Based Battery Anode Material with Exceptional High-Temperature Cycling Stability. Nano Energy.

[89] Ghaani M.R., Mohtasebi A.M., Tajeri R., Marashi P. (2020). A Comparison of the Role of the Chelating Agent on the Structure of Lithium Conducting Solid Electrolyte Li_1.4_Al_0.4_Ti_1.6_(PO_4_)_3_: Pechini vs. Modified Pechini-Type Methods. Batteries.

[90] Peng J., Li W., Wu Z., Li H., Huang Y., Ouyang Y., Wang Y., Guo X., Chen G., Wang X. (2021). Rational Design and Performance of Ansode Materials Based on Si/SiO_x_/C Particles Anchored on Graphene Sheets. ACS Appl. Energy Mater..

[91] Shao G., Hanaor D.A.H., Wang J., Kober D., Li S., Wang X., Shen X., Bekheet M.F., Gurlo A. (2020). Polymer-Derived SiOC Integrated with a Graphene Aerogel As a Highly Stable Li-Ion Battery Anode. ACS Appl. Mater. Interfaces.

[92] Park M., Park E., Lee J., Jeong G., Kim K.J., Kim J.H., Kim Y., Kim H. (2014). Hydrogen Silsequioxane-Derived Si/SiO. ACS Appl. Mater. Interfaces.

[93] Yoo H., Park E., Bae J., Lee J., Chung D.J., Jo Y.N., Park M.S., Kim J.H., Dou S.X., Kim Y.J. (2018). Si Nanocrystal-Embedded SiO_x_ Nanofoils: Two-Dimensional Nanotechnology-Enabled High Performance Li Storage Materials. Sci. Rep..

[94] Zheng G., Xiang Y., Xu L., Luo H., Wang B., Liu Y., Han X., Zhao W., Chen S., Chen H. (2018). Controlling Surface Oxides in Si/C Nanocomposite Anodes for High-Performance Li-Ion Batteries. Adv. Energy Mater..

[95] Wu H., Yu G., Pan L., Liu N., McDowell M.T., Bao Z., Cui Y. (2013). Stable Li-Ion Battery Anodes by in-Situ Polymerization of Conducting Hydrogel to Conformally Coat Silicon Nanoparticles. Nat. Commun..

[96] Zhang T., Gao J., Zhang H.P., Yang L.C., Wu Y.P., Wu H.Q. (2007). Preparation and electrochemical properties of core-shell Si/SiO nanocomposite as anode material for lithium-ion batteries. Electrochem. Commun..

[97] Frangieh C.J., Fan J.L., Melms J.C., Shah P., Azizi E., Izar B. (2026). Single-Cell and Spatial Profiling in Cancer Biology and Clinical Oncology. Nat. Cancer.

[98] Liu Z., Zhao Y., He R., Luo W., Meng J., Yu Q., Zhao D., Zhou L., Mai L. (2019). Yolk@Shell SiO_x_/C microspheres with semi-graphitic carbon coating on the exterior and interior surfaces for durable lithium storage. Energy Storage Mater..

[99] Luo W., Wang Y., Chou S., Xu Y., Li W., Kong B., Dou S.X., Liu H.K., Yang J. (2016). Critical thickness of phenolic resin-based carbon interfacial layer for improving long cycling stability of silicon nanoparticle anodes. Nano Energy.

[100] Ge G., Li G., Wang X., Chen X., Fu L., Liu X., Mao E., Liu J., Yang X., Qian C. (2021). Manipulating Oxidation of Silicon with Fresh Surface Enabling Stable Battery Anode. Nano Lett..

[101] Devarapalli R.R., Szunerits S., Coffinier Y., Shelke M.V., Boukherroub R. (2016). Glucose-Derived Porous Carbon-Coated Silicon Nanowires as Efficient Electrodes for Aqueous Micro-Supercapacitors. ACS Appl. Mater. Interfaces.

[102] Gao S., Yang D., Pan Y., Geng L., Li S., Li X., Cao P.F., Yang H. (2019). From Natural Material to High-Performance Silicon Based Anode: Towards Cost-Efficient Silicon Based Electrodes in High-Performance Li-Ion Batteries. Electrochim. Acta.

[103] Mokoena E.M., Datye A.K., Coville N.J. (2003). A Systematic Study of the Use of DL-Tartaric Acid in the Synthesis of Silica Materials Obtained by the Sol-Gel Method. J. Sol-Gel Sci. Technol..

[104] Dirican M., Yanilmaz M., Fu K., Yildiz O., Kizil H., Hu Y., Zhang X. (2014). Carbon-Confined PVA-Derived Silicon/Silica/Carbon Nanofiber Composites as Anode for Lithium-Ion Batteries. J. Electrochem. Soc..

[105] Luo W., Wang Y., Wang L., Jiang W., Chou S.L., Dou S.X., Liu H.K., Yang J. (2016). Silicon/Mesoporous Carbon/Crystalline TiO_2_ Nanoparticles for Highly Stable Lithium Storage. ACS Nano.

[106] Liu Y., Qin L., Liu F., Fan Y., Ruan J., Zhang S. (2018). Interpenetrated 3D Porous Silicon as High Stable Anode Material for Li-Ion Battery. J. Power Sources.

[107] Cook J.B., Kim H.S., Lin T.C., Robbennolt S., Detsi E., Dunn B.S., Tolbert S.H. (2017). Tuning Porosity and Surface Area in Mesoporous Silicon for Application in Li-Ion Battery Electrodes. ACS Appl. Mater. Interfaces.

[108] Yang Y., Yang X., Chen S., Zou M., Li Z., Cao A., Yuan Q. (2017). Rational Design of Hierarchical Carbon/Mesoporous Silicon Composite Sponges as High-Performance Flexible Energy Storage Electrodes. ACS Appl. Mater. Interfaces.

[109] Zuo X., Wen Y., Qiu Y., Cheng Y.J., Yin S., Ji Q., You Z., Zhu J., Müller-Buschbaum P., Ma L. (2020). Rational Design and Mechanical Understanding of Three-Dimensional Macro-/Mesoporous Silicon Lithium-Ion Battery Anodes with a Tunable Pore Size and Wall Thickness. ACS Appl. Mater. Interfaces.

[110] Zuo X., Xia Y., Ji Q., Gao X., Yin S., Wang M., Wang X., Qiu B., Wei A., Sun Z. (2017). Self-Templating Construction of 3D Hierarchical Macro-/Mesoporous Silicon from 0D Silica Nanoparticles. ACS Nano.

[111] Zhang X., Huang L., Zeng P., Wu L., Zhang R., Chen Y. (2018). Highly Porous Si Nanoframeworks Stabilized in TiO_2_ Shells and Enlaced by Graphene Nanoribbons for Superior Lithium-Ion Storage. ChemElectroChem.

[112] Zhang F.Z., Ma Y.Y., Jiang M.M., Luo W., Yang J.P. (2022). Boron Heteroatom-Doped Silicon–Carbon Peanut-like Composites Enables Long Life Lithium-Ion Batteries. Rare Met..

[113] Luo W., Shen D., Zhang R., Zhang B., Wang Y., Dou S.X., Liu H.K., Yang J. (2016). Germanium Nanograin Decoration on Carbon Shell: Boosting Lithium-Storage Properties of Silicon Nanoparticles. Adv. Funct. Mater..

[114] Liu T., Dong T., Wang M., Du X., Sun Y., Xu G., Zhang H., Dong S., Cui G. (2024). Recycled Micro-Sized Silicon Anode for High-Voltage Lithium-Ion Batteries. Nat. Sustain..

[115] Yi R., Gordin M.L., Wang D. (2016). Integrating Si Nanoscale Building Blocks into Micro-Sized Materials to Enable Practical Applications in Lithium-Ion Batteries. Nanoscale.

[116] Wild J.F., Li Y., Zhou Y., Bao W., Gujarathi A., Zhang R., Yang Y., Hu T., Jiang T., Chen Y. (2025). Micron-Sized Nanostructured Si−C Composites for High Performance Li-Ion Battery Anodes. ACS Appl. Nano Mater..

[117] Niu J., Lee J.Y. (2002). Improvement of Usable Capacity and Cyclability of Silicon-Based Anode Materials for Lithium Batteries by Sol-Gel Graphite Matrix. Electrochem. Solid-State Lett..

[118] Chen W.-T., Muruganantham R., Liu W.-R. (2022). Construction of 3D porous graphene aerogel wrapped silicon composite as anode materials for high-efficient lithium-ion storage. Surf. Coat. Technol..

[119] Aghamohammadi H., Torabian M. (2026). Advances in 3D Si-graphene nanocomposites for Li-ion battery anodes with enhanced stability and rate capability. Particuology.

[120] Gurav J.L., Jung I.-K., Park H.-H., Kang E.S., Nadargi D.Y. (2010). Silica Aerogel: Synthesis and Applications. J. Nanomater..

[121] Patil R., Phadatare M., Blomquist N., Örtegren J., Hummelgård M., Meshram J., Dubal D., Olin H. (2021). Highly Stable Cycling of Silicon-Nanographite Aerogel-Based Anode for Lithium-Ion Batteries. ACS Omega.

[122] Phadatare M., Patil R., Blomquist N., Forsberg S., Örtegren J., Hummelgård M., Meshram J., Hernández G., Brandell D., Leifer K. (2019). Silicon-Nanographite Aerogel-Based Anodes for High Performance Lithium Ion Batteries. Sci. Rep..

[123] Hu X., Jin Y., Zhu B., Tan Y., Zhang S., Zong L., Lu Z., Zhu J. (2016). Free-Standing Graphene-Encapsulated Silicon Nanoparticle Aerogel as an Anode for Lithium-Ion Batteries. ChemNanoMat.

[124] Jeong G., Kim J.-H., Kim Y.-U., Kim Y.-J. (2012). Multifunctional TiO_2_ coating for a SiO anode in Li-ion batteries. J. Mater. Chem..

[125] Zhu H., Shiraz M.H.A., Liu L., Hu Y., Liu J. (2021). A facile and low-cost Al_2_O_3_ coating as an artificial solid electrolyte interphase layer on graphite/silicon composites for lithium-ion batteries. Nanotechnology.

[126] Deng W., Ni S., Hu N., Zhou Y., Jin Z. (2024). SiO_x_@ZrO_2_@C Nanospheres as a High-Capacity and Stable Anode Material for Lithium-Ion Batteries. ACS Appl. Nano Mater..

[127] Choi D., Choy K.-L. (2016). Novel Nanostructured SiO_2_/ZrO_2_ Based Electrodes with Enhanced Electrochemical Performance for Lithium-ion Batteries. Electrochem. Acta.

[128] Yang J., Wang Y., Li W., Wang L., Fan Y., Jiang W., Luo W., Wang Y., Kong B., Selomulya C. (2017). Amorphous TiO_2_ Shells: A Vital Elastic Buffering Layer on Silicon Nanoparticles for High-Performance and Safe Lithium Storage. Adv. Mater..

[129] Nazir S., Li W., Ishtiaq B., Rubab R., Batool K. (2026). Titanium Oxide-Based Anodes for Lithium Batteries Transitioning from Liquid-Phase Studies to Solid-State Integration. Coord. Chem. Rev..

[130] Fang S., Bresser D., Passerini S. (2020). Transition Metal Oxide Anodes for Electrochemical Energy Storage in Lithium- and Sodium-Ion Batteries. Adv. Eng. Mater..

[131] Petrovičovà B., Xu W., Musolino M.G., Pant F., Patan S., Santangelo S., Triolo C., Pinna N. (2022). High-Entropy Spinel Oxides Produced via Sol-Gel and Electrospinning and Their Evaluation as Anodes in Li-Ion Batteries. Appl. Sci..

[132] Yang X., Wang H., Song Y., Liu K., Huang T., Wang X., Zhang C., Li J. (2022). Low-Temperature Synthesis of a Porous High-Entropy Transition- Metal Oxide as an Anode for High-Performance Lithium-Ion Batteries. ACS Appl. Mater. Interfaces.

[133] Islam M., Ahmed S., Faizan M., Ali B., Bhuyan M., Bari G.A.K.M.R., Nam K. (2024). Review on the Polymeric and Chelate Gel Precursor for Li-Ion Battery Cathode Material Synthesis. Gels.

[134] Mathew V., Sambandam B., Kim S., Kim S., Park S., Lee S., Lee J., Park S., Song J., Kim J. (2020). High-voltage cathode materials by combustion-based preparative approaches for Li-ion batteries application. J. Power Sources.

[135] Danks A.E., Hall S.R., Schnepp Z. (2016). The evolution of ‘sol–gel’ chemistry as a technique for materials synthesis. Mater. Horiz..

[136] Zakharova G.S., Singer L., Fattakhova Z.A., Wegener S., Thauerb E., Zhu Q., Shalaeva E.V., Klingeler R. (2021). MoO_2_/C composites prepared by tartaric acid and glucose-assisted sol-gel processes as anode materials for lithium-ion batteries. J. Alloys Comp..

[137] Thauer E., Zakharova G.S., Wegener S.A., Zhu Q., Klingeler R. (2021). Sol-gel synthesis of Li_3_VO_4_/C composites as anode materials for lithium-ion batteries. J. Alloys Comp..

[138] Singh G., Sil A., Ghosh S., Panwar A. (2010). Effect of citric acid content on synthesis of LiNi_1/3_Mn_1/3_Co_1/3_O_2_ and its electrochemical characteristics. Ceram. Int..

[139] Wang C., Chen X., Wang B., Huang M., Wang B., Jiang Y., Ruoff R.S. (2018). Freeze-Casting Produces a Graphene Oxide Aerogel with a Radial and Centrosymmetric Structure. ACS Nano.

[140] He J., Zhang H., Zhao J., Zhou L., Luo C., Li X., Hu Y., Liu Y., Yang D., Cui X. (2023). Optimizing solution combustion method fuel ratios for enhanced electrochemical performance of LiNi_0.5_Mn_1.5_O_4_ cathode materials. J. Alloys Comp..

[141] Guo J., Chen L., Zhang X., Jiang B., Ma L. (2014). Sol-Gel Synthesis of Mesoporous Co_3_O_4_ Octahedra toward High-Performance Anodes for Lithium-Ion Batteries. Electrochim. Acta.

[142] Sun C., Chen S., Li Z. (2018). Controllable Synthesis of Fe_2_O_3_-Carbon Fiber Composites via a Facile Sol-Gel Route as Anode Materials for Lithium Ion Batteries. Appl. Surf. Sci..

[143] Lv P., Zhao H., Zeng Z., Wang J., Zhang T., Li X. (2014). Facile Preparation and Electrochemical Properties of Carbon Coated Fe_3_O_4_ as Anode Material for Lithium-Ion Batteries. J. Power Sources.

[144] Guo S., Ait M., Li J., Wu J., Shi X., Pao C., Hu Z., Ma J. (2025). Fluorine Doping Induced Accelerated Li^+^ Kinetics in Conversion-Type Fe_3_O_4_ Anode. J. Power Sources.

[145] Alam M.W., Baqais A., Rahman M.M., Aamir M., Abuzir A., Mushtaq S., Amin M.N., Khan M.S. (2022). Investigation on In Situ Carbon-Coated ZnFe_2_O_4_ as Advanced Anode Material for Li-Ion Batteries. Gels.

[146] Cai K., Luo S., Cong J., Li K., Yan S., Hou P., Song Y., Wang Q., Zhang Y., Liu X. (2022). Sol-Gel Synthesis of Nano Block-like ZnMn_2_O_4_ Using Citric Acid Complexing Agent and Electrochemical Performance as Anode for Lithium-Ion Batteries. J. Alloys Compd..

[147] Xu Y., Hong Z., Xia L., Yang J., Wei M. (2013). One Step Sol–Gel Synthesis of Li_2_ZnTi_3_O_8_/C Nanocomposite with Enhanced Lithium-Ion Storage Properties. Electrochim. Acta.

[148] Ma Y. (2020). Sol-Gel Synthesis of ZnO/TiO_2_ Core-Shell Nanocomposites and Their Structural and Electrochemical Characterization as Anode for Lithium Ion Battery. Int. J. Electrochem. Sci..

[149] Osman A., Akbulut H.A. (2015). Facile Synthesis of Zinc Oxide/Multiwalled Carbon Nanotube Nanocomposite Lithium-Ion Battery Anodes by Sol-Gel Method. J. Power Sources.

[150] Zhang C., Zhang Y., Wang J., Wang D., He D., Xia Y. (2013). Li_4_Ti_5_O_12_ Prepared by a Modified Citric Acid Sol-Gel Method for Lithium-Ion Battery. J. Power Sources.

[151] Kuo Y., Lin J. (2014). One-Pot Sol-Gel Synthesis of Li_4_Ti_5_O_12_/C Anode Materials for High-Performance Li-Ion Batteries. Electrochim. Acta.

[152] Lee S., Cho I., Lee J.H., Kim D.H., Kim D.W., Kim J.Y., Shin H., Lee J., Jung O.H.S., Park N.-G. (2010). Two-Step Sol-Gel Method-Based TiO_2_ Nanoparticles with Uniform Morphology and Size for Efficient Photo-Energy Conversion Devices. Chem. Mater..

[153] Nian J., Teng H. (2006). Hydrothermal Synthesis of Single-Crystalline Anatase TiO_2_ Nanorods with Nanotubes as the Precursor. J. Phys. Chem. B.

[154] Wu C., Wang Y., Ma G., Zheng X. (2021). Electrochemistry Communications Enhanced Rate Capability of Li_4_Ti_5_O_12_ Anode Material by a Photo-Assisted Sol–Gel Route for Lithium-Ion Batteries. Electrochem. Commun..

[155] Islam M., Ali G., Jeong M.-G., Choi W., Chung K.Y., Jung H.-G. (2017). Study on the Electrochemical Reaction Mechanism of NiFe_2_O_4_ as a High-Performance Anode for Li-Ion Batteries. ACS Appl. Mater. Interfaces.

[156] Chchiyai Z., Ghali O.E., Lahmar A., Alami J., Manoun B. (2023). Design and Performance of a New Zn_0.5_Mg_0.5_FeMnO_4_ Porous Spinel as Anode Material for Li-Ion Batteries. Molecules.

[157] Zhu C., Han C.-G., Saito G., Akiyama T. (2016). Facile synthesis of MnO/carbon composites by a single-step nitrate-cellulose combustion synthesis for Li ion battery anode. J. Alloys Comp..

[158] Han C.-G., Zhu C., Aoki Y., Habazaki H., Akiyama T. (2017). MnO/N–C anode materials for lithium-ion batteries prepared by cotton-templated combustion synthesis. Green Energy Environ..

